# Molecular plasticity of herpesvirus nuclear egress analysed *in situ*

**DOI:** 10.1038/s41564-024-01716-8

**Published:** 2024-06-25

**Authors:** Vojtěch Pražák, Yuliia Mironova, Daven Vasishtan, Christoph Hagen, Ulrike Laugks, Yannick Jensen, Saskia Sanders, John M. Heumann, Jens B. Bosse, Barbara G. Klupp, Thomas C. Mettenleiter, Michael Grange, Kay Grünewald

**Affiliations:** 1 Centre for Structural Systems Biology, 22607 Hamburg, Germany; 2 Leibniz Institute of Virology, 20251 Hamburg, Germany; 3 Oxford Particle Imaging Centre, Division of Structural Biology, Wellcome Trust Centre for Human Genetics, University of Oxford, Roosevelt Drive, Oxford OX3 7BN, UK; 4 Department of Biochemistry, University of Oxford, South Parks Road, Oxford, OX1 3QU; 5 Department of Chemistry, University of Hamburg, 20146, Hamburg, Germany; 6 Hannover Medical School, Institute of Virology, 30625 Hannover, Germany; 7 Cluster of Excellence RESIST (EXC 2155), Hannover Medical School, 30625 Hannover, Germany; 8 Molecular, Cellular and Developmental Biology, University of Colorado, Boulder, USA; 9 Institute of Molecular Virology and Cell Biology, Friedrich-Loeffler-Institut, Greifswald-Insel Riems, Germany; 10 Structural Biology, Rosalind Franklin Institute, Harwell Campus, Didcot, OX11 0FA, United Kingdom

## Abstract

The viral nuclear egress complex (NEC) allows herpesvirus capsids to escape from the nucleus without compromising the nuclear envelope integrity.

The NEC lattice assembles on the inner nuclear membrane and mediates the budding of nascent nucleocapsids into the perinuclear space and their subsequent release into the cytosol. Its essential role makes it a potent antiviral target, necessitating structural information in the context of a cellular infection. Here we determined structures of NEC–capsid interfaces in situ using electron cryo-tomography, showing a substantial structural heterogeneity. In addition, while the capsid is associated with budding initiation, it is not required for curvature formation. By determining the NEC structure in several conformations, we show that curvature arises from an asymmetric assembly of disordered and hexagonally ordered lattice domains independent of pUL25 or other viral capsid vertex components. Our results advance our understanding of the mechanism of nuclear egress in the context of a living cell.

## Introduction

Herpesvirus virions consist of icosahedral capsids, enclosing a DNA genome, embedded in a tegument layer and an envelope. Capsid assembly takes place in the nucleus; it results in the formation of three distinct forms referred to as A-, B- and C-capsids (empty, scaffold-containing and DNA-containing capsids, respectively)^1^. The newly formed 125 nm diameter particles exceed the ~40 nm size exclusion limit of the nuclear pore complex^2^ and are instead transported via a specialised mechanism involving membrane envelopment/de-envelopment termed herpesvirus nuclear egress^3,4^. Firstly, progeny nucleocapsids are enveloped at the inner nuclear membrane (INM) and bud into the perinuclear space. The resulting primary enveloped virions (nucleocapsids inside perinuclear vesicles) then fuse with the outer nuclear membrane (ONM), releasing the nucleocapsids into the cytosol for subsequent secondary envelopment and egress^3,5^.

The main mediators of nuclear egress in alphaherpesviruses, including pseudorabies virus (PrV) and herpes simplex virus-1 (HSV-1), are homologues of pUL31 and pUL34 (Extended Data Table 1), which form heterodimers known as the nuclear egress complex^6^ (NEC; for comparison of relevant herpesvirus gene names see Extended Data Table 1^7^). The NEC self-associates at the INM, forming a hexameric lattice capable of inducing membrane curvature. Canonically, budding occurs upon association with a nucleocapsid^6,8,9^. However, the NEC also forms perinuclear vesicles in the absence of any other viral proteins^10–12^. As such, the mechanism of nucleocapsid recruitment by the NEC remains ambiguous. There are a number of candidates for the physical interaction between nucleocapsid and the NEC, chiefly pUL17 and pUL25^13–15^. Even though pUL25 is not strictly required for egress^16^, these two proteins together with pUL36 form a structural component of the nucleocapsid termed the capsid-vertex specific component (CVSC) ^17–19^. The CVSC rests on the fivefold vertex of the nucleocapsid of herpesviruses^20,21^. It is generally assumed to mediate the selection of fully formed capsids for egress^22^, although a molecular scale description for this is lacking. To date, integrating results from reconstituted systems has been the primary means of understanding this process, with the necessary in situ data lacking^23,24^.

In the present study, we visualise all stages of nuclear egress in the context of frozen hydrated cells infected by PrV-ΔUS3, wild-type (WT) PrV, or WT HSV-1. First, using a combination of cryoET and subvolume averaging we explore the structures involved in the initiation of envelopment. Contrary to our current understanding, the CVSC does not mediate the capsid-NEC interaction as it is only fully assembled in the cytosol. We then show that the curvature in spherical NEC-coated vesicles is generated through the formation of both hexagonally ordered and disordered regions. Furthermore, by comparing NEC lattices with different curvatures, we show that the core hexameric repeating unit is highly flexible and capable of taking on less symmetrical forms. Together, we present a holistic structural view of herpesvirus nuclear egress and a framework for integrating targeted perturbations of this transport system.

## Results

### CryoET reveals Pseudorabies virus nuclear egress in situ

**Extended Data Figure 1:**
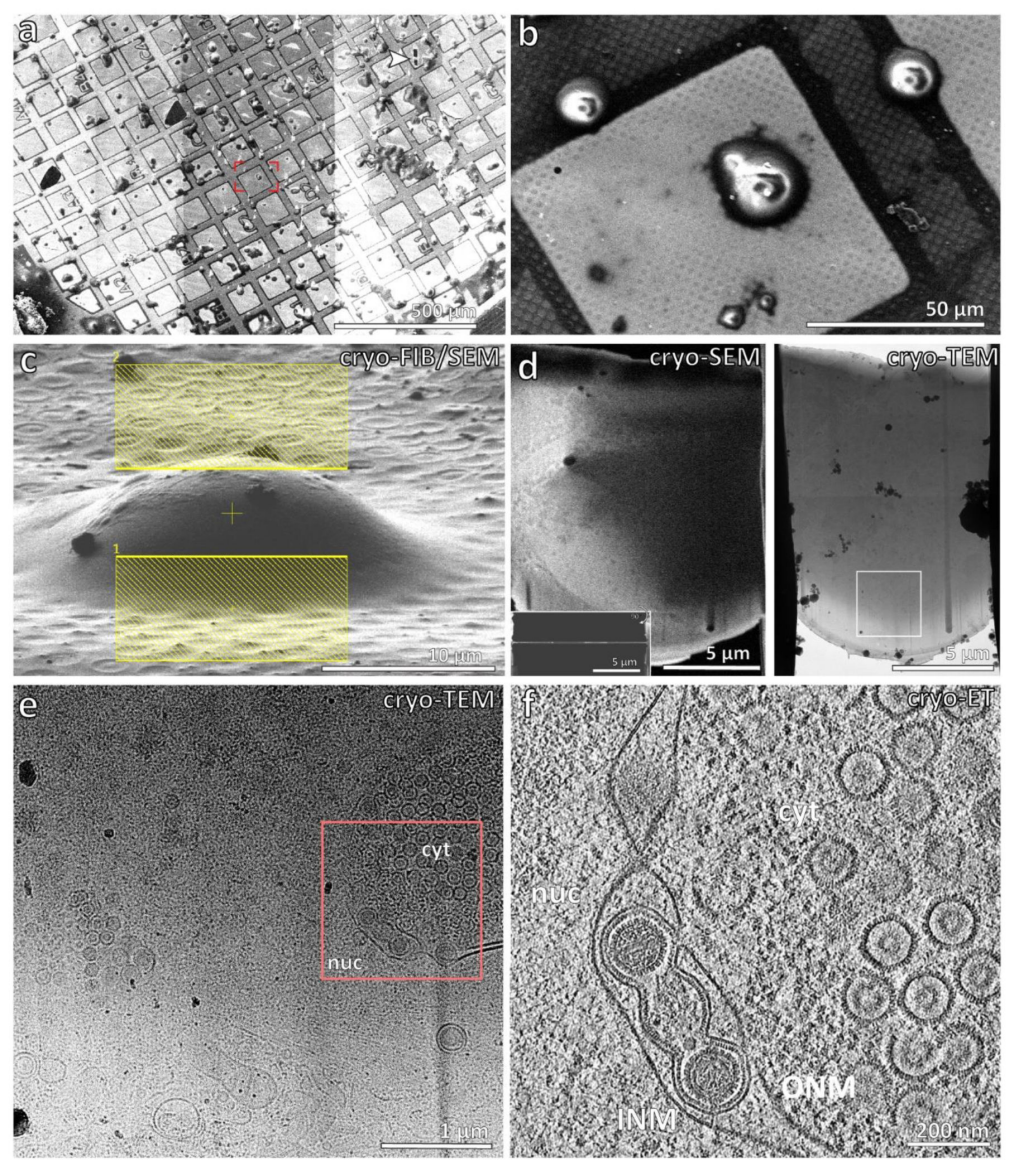
Workflow for CryoFIB/SEM and CryoET. **a**, A low magnification SEM image of PrV-ΔUS3-infected porcine epithelial cells. **b**, A higher magnification image of the box shown in a. **c**, An oblique SEM view illuminated by the FIB with yellow boxes indicating the area above and below the cell for targeting with the focused ion beam. **d**, SEM image of thinned cellular section (lamella) from the top and side (inset). Transmission electron microscope (TEM) image is also shown (right), with target area shown in e highlighted by a white box. **e**, A TEM image shown at 9500x nominal magnification. Details of the cell are visible at this magnification, allowing targeting of regions of interest for higher magnification tomographic data collection (red box). Shown are representative samples of 113 lamella from 3 biological replicates. cyt = cytosol, nuc = nucleus. **f**, A tomographic slice of the region indicated in e) at 35000x magnification, nominal. ONM, outer nuclear membrane; INM, inner nuclear membrane.

**Extended Data Fig. 2:**
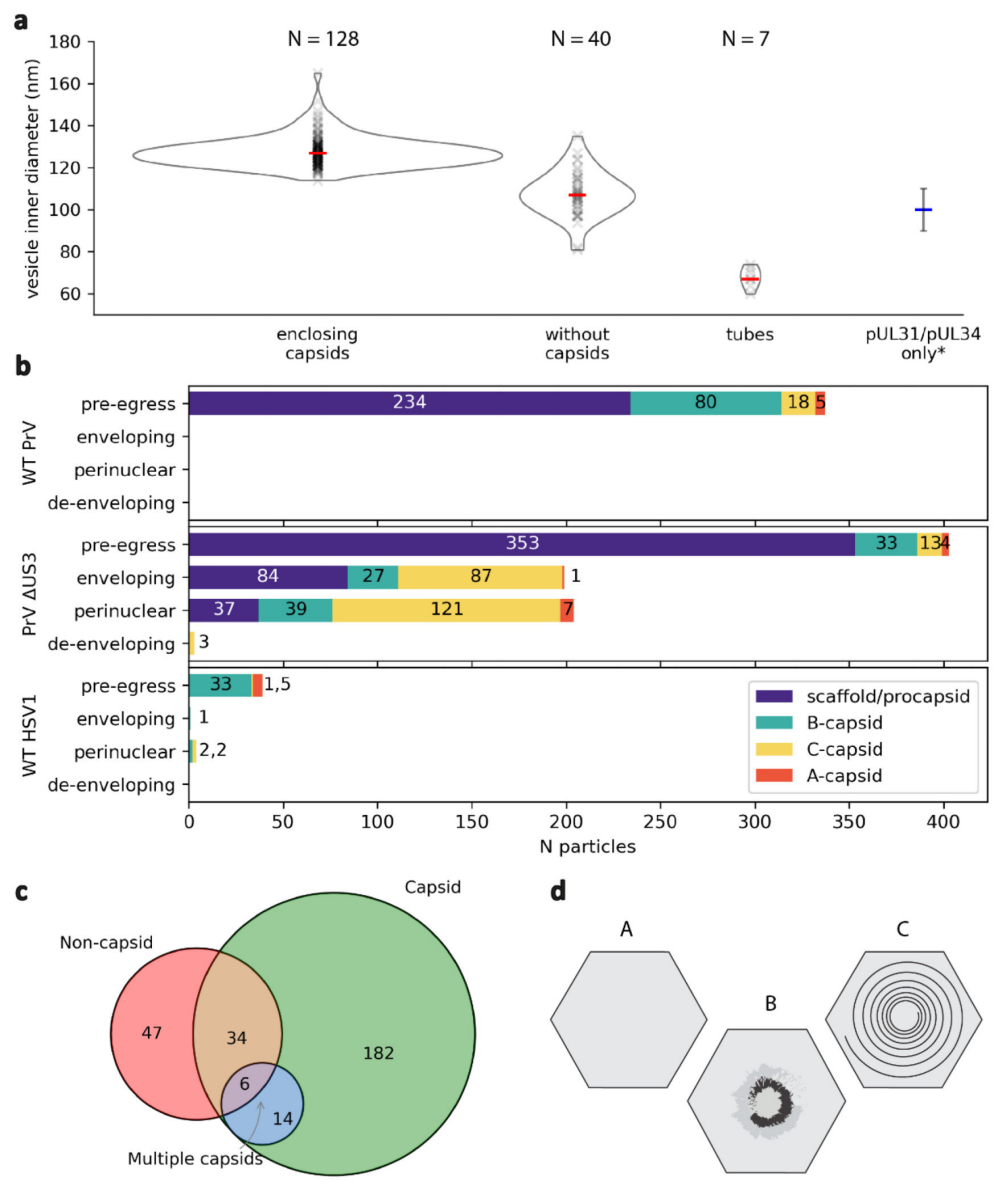
Quantification of nuclear egress events. **a**, Size and curvature distribution of perinuclear vesicles. Shown are inner luminal diameters of individual vesicles (see Methods) with mean values represented by red bars. *The mean value and standard deviation of perinuclear vesicles obtained by expression pUL31/pUL34^9^ are shown for comparison. Vesicles (and the cargo within) were counted as perinuclear where envelopment was judged to be more than 50% completed (for example, Fig. 1c would be considered perinuclear). This is because a significant proportion of vesicles (~150 nm diameter) were only partially contained within the 150-250 nm thick tomograms (that is, cut off during FIB-milling). **b**, Quantification of capsid types observed during nuclear egress in porcine epithelial (PrV) and African Green monkey cells (HSV-1). **c**, Quantification of perinuclear vesicles. Classes were assigned to vesicles based on cargo type. In contrast to vesicle diameter measurements shown in **a**, vesicles containing procapsids were included in the capsid class, whereas scaffolds were counted as non-capsid cargo (note that there were 6 perinuclear procapsids). Procapsids contain capsid proteins some of which could be involved in the initiation of envelopment and therefore fit better in the “capsid” class. At the same time, all perinuclear procapsids were only partially assembled and consequently the perinuclear vesicles were smaller. **d**, Diagrams indicating capsid classification. A-capsids lack both nucleic acid and scaffold, B-capsids contain a scaffold, and C-capsids are capsids after completion of DNA packaging. Procapsids (not shown) are C-capsid precursors.

**Fig. 1:**
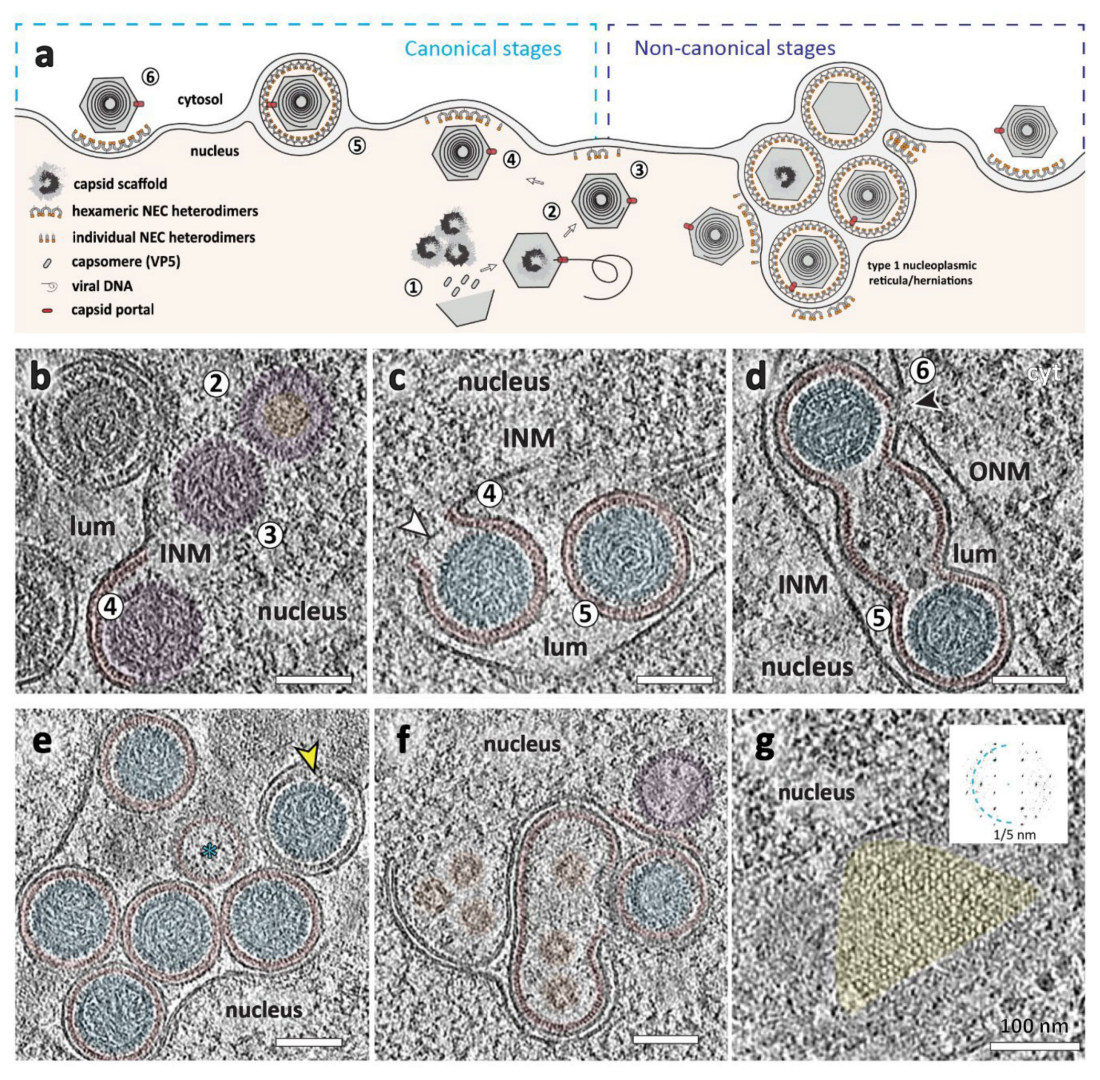
Visualising stages of PrV nuclear egress. **a**, Schematic model of capsid assembly and nuclear egress via primary envelopment. Numbers indicate (selected) consecutive stages of assembly and nuclear egress. Canonically, the NEC envelops nucleocapsids (capsids with DNA) and forms individual spherical perinuclear vesicles. Other structures (for example, bundles of perinuclear vesicles) are considered non-canonical. **a-g**, The NEC is highlighted in red, nuclear capsids in purple and perinuclear capsids in blue. **b**, CryoET slice of stages 2 (pro-/B-capsid) and 3 (nuclear C-capsid) leading up to 4, the interaction of the nuclear C-capsid with the NEC at the INM. **c**, Budding of the nucleocapsid into the perinuclear space (lumen between nuclear membranes) to form primary-enveloped particles (stages 4 and 5). The white arrow indicates the membrane opening to the nucleus. **d**, Primary enveloped capsids in the lumen and perinuclear space traversing across the INM and ONM (stage 5 and 6). The black arrow indicates a membrane of the primary vesicle open to the cytosol. **e**, Perinuclear vesicles inside a large type-1 nucleoplasmic reticulum within the lumen between the INM and the ONM. The cyan asterisk indicates an empty (that is, lacking assembled capsid components) perinuclear vesicle; note that the slicing plane does not go through the vesicle equator. The yellow arrow indicates an example of a vesicle with a gap between the NEC and capsid. **f**, A perinuclear vesicle and an INM infolding containing several procapsid scaffolds (orange). **g**, Top view of a putative head-head stacked double NEC layer (yellow). The inset shows the power spectrum (frequency space representation indicating the periodicity of components) of a projection through the lattice layer, indicating a hexagonal lattice spacing. Scale bars are 100 nm. ONM = outer nuclear membrane, INM = inner nuclear membrane, lum = lumen/perinuclear space. The data presented here are from 32 tomograms and 3 biological replicates.

We used focused ion beam and scanning electron cryo-microscopy (cryoFIB-SEM) together with electron cryo-tomography (cryoET) to directly view herpesvirus capsid assembly and nuclear egress in porcine cells infected with PrV-ΔUS3 (Fig. 1, Extended Data Fig. 1, Supplementary Video 1). In the PrV-ΔUS3 system^25–28^, primary enveloped (perinuclear) particles accumulate in the perinuclear space, enabling sufficient volumes of data to be acquired for high resolution structural analyses. The 10-nm thick NEC coat could be observed in association with nucleocapsids at the INM at different stages of envelopment, from initial nucleocapsid-NEC contact, to partially coated capsids, and almost fully formed vesicles connected to the nucleoplasm through a narrow pore (Fig. 1c; stages quantified in Extended Data Fig. 2b). Fully formed perinuclear vesicles, roughly spherical in shape, constituted the majority of the NEC lattice occurrences in our data. The mean inner diameter of isolated perinuclear vesicles containing single capsids was 128 ± 7 nm, (range represents standard deviation, N = 128, Extended Data Fig. 2). Finally, there were perinuclear vesicles partially opened to the cytosol with fusion pores visible in the ONM, exposing the NEC coat to the cytosolic environment (N = 2). Interestingly, the NEC lattice remained intact, while the ONM was clearly open to the cytosol (Fig. 1d, Extended Data Fig. 1, Supplementary Video 2).

**Extended Data Fig. 3:**
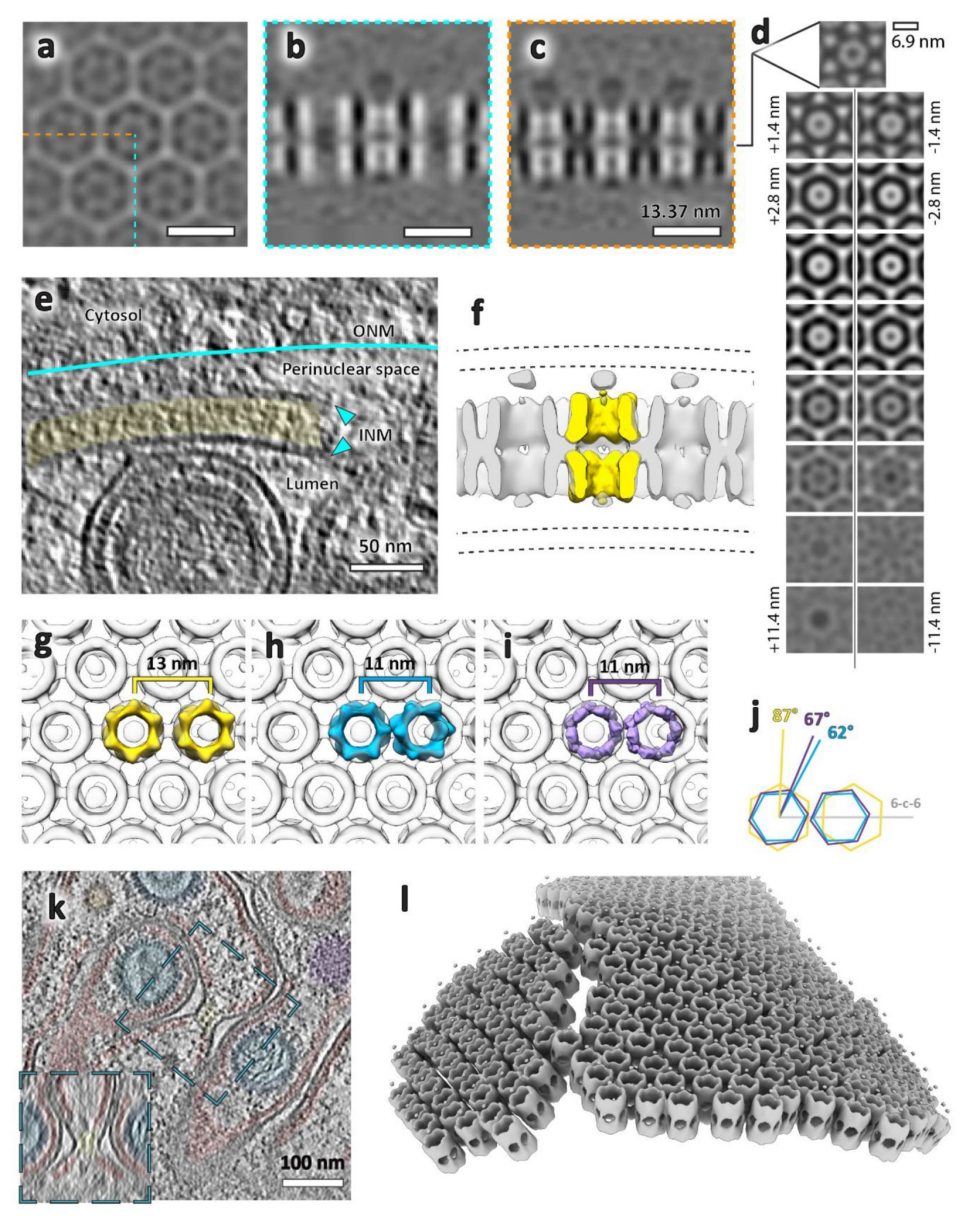
Putative flat NEC double-layer lattice. **a, b, c** Orthogonal section through average volume of lattice shown in Fig. 1f. There is no density for the membrane bilayers due to the majority of particles having their six-fold symmetry aligned with the tomogram Z axis (and the missing wedge). A total of 1944 C6 symmetrised particles were included in the average. **d**, Tangential sections through the volume at 1.4 nm intervals. **e**, A slice through the raw tomogram indicating the position of the inner nuclear membranes relative to the lattice. The membrane is not visible around the majority of the lattice layer due to its orientation to the tomogram missing wedge. The approximate position of the outer nuclear membrane was inferred from the exclusion zone of cytosolic components (ribosomes, intermediate filaments, microtubules). **f**, Two NEC hexamers segmented from the spherical lattice (Fig. 5, here shown in yellow) were fitted into one repeating unit of the lattice. In this orientation, pUL31 would form the interface between the two lattice layers. **g, h, i, j**, The hexamer centres in the double-layer lattice (yellow) are spaced 2 nm further apart and are rotated by approximately 20° to the 6-2-6 axis compared to spherical NEC (blue) and flat lattice derived from the HSV-1 crystal structure (purple). **k**, Slice through several adjacent type-1 NR, with the lumen of one of these zippered by putative head-head interacting NEC (yellow). Inset shows an orthogonal slice through the centre of the highlighted area. **i**, Plotback of individual symmetry units (dodecamers). There is a slight curvature to the lattice with a break in the middle, presumably to accommodate the tighter curvature of the underlying nucleoplasmic reticulum membrane.

Apart from singular, well-formed primary virions which had budded individually into the perinuclear lumen, we observed type-1 nucleoplasmic reticula-like^29^ (NR) structures containing bundles of perinuclear vesicles, similar to previously described herniations^30,31^ (Fig. 1e, Supplementary Video 3). We could also observe type-2 NR, in which invaginations of both INM and ONM allowed the close proximity of cytosol to the centre of nuclei^29^ (Extended Data Fig. 1e). Perinuclear vesicles in type-1 NR had a number of morphologies (quantified in Extended Data Fig. 2): spherical vesicles containing capsids, smaller vesicles containing non-capsid cargo such as capsid scaffolds or partially assembled procapsids (Fig. 1e, 107 ± 11 nm SD diameter, N = 40), ellipsoidal vesicles containing multiple capsids and non-capsid cargos (Fig. 1f), multi-chambered vesicles (Fig. 1d), and even elongated tubular structures, often devoid of identifiable cargo (Supplementary Video 4). Finally, there were two instances of NEC lattice interacting head to head and even forming flat, double-layer sheets (Fig. 1g, Extended Data Fig. 3). These observations show that the NEC is capable of forming a variety of distinct structures depending on the molecular context and can transport capsids at different stages of assembly.

### NEC-capsid contacts are pleomorphic

**Extended Data Fig. 4:**
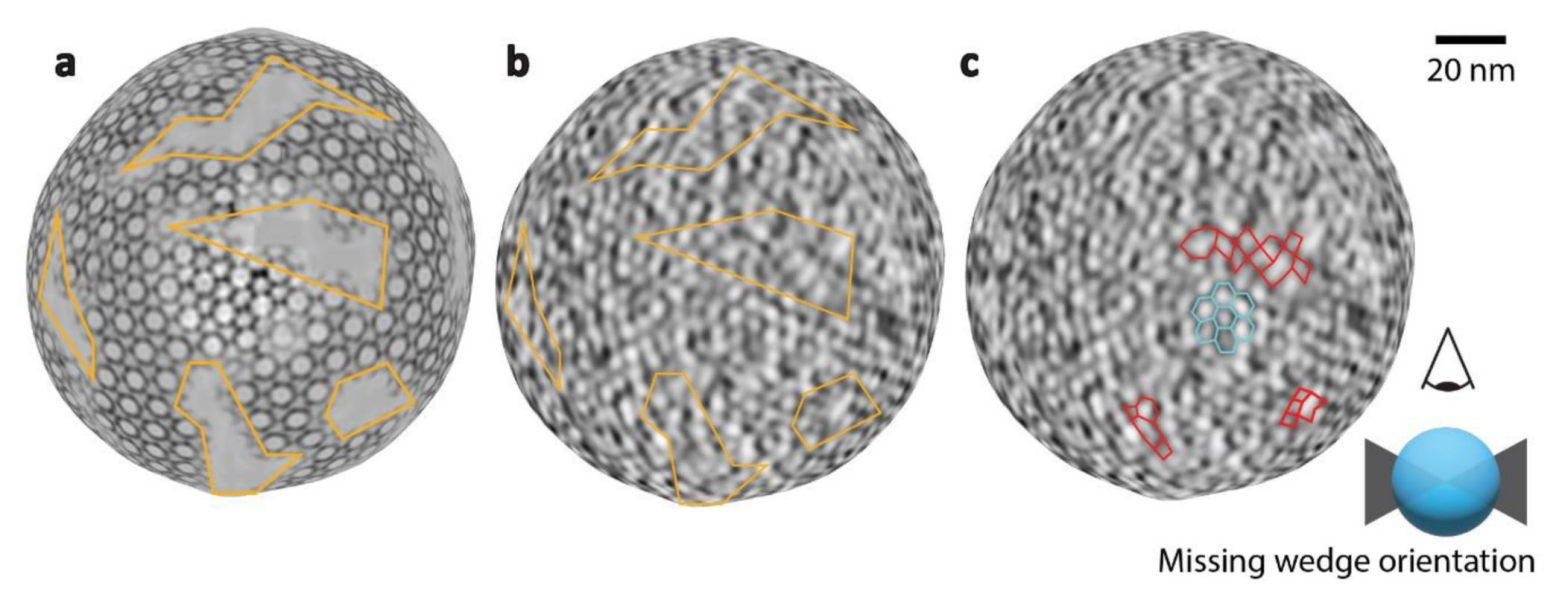
Subvolume averaging generates a plausible model of NEC lattice order. Shown is a top view of a single perinuclear vesicle (also in Figs. 2, 3, and Supplementary Fig. 8). The surface (generated and visualised using Open3D) was coloured with the intersecting voxel densities of either **a**, volume where an NEC average volume was backplotted using subvolume averaging particle positions or **b, c**, the original data. Orange lines highlight the same areas in a and b where particles were removed due to their relatively low cross correlation coefficient. A long-range hexagonal order is apparent outside these areas. Assessing the nature of disordered regions is more challenging. To guide the reader’s eye, some densities in putative disordered regions were highlighted with red lines. A single hexagonal region was highlighted in blue for comparison. Note: A direct interpretation of tomogram densities on this scale can be misleading and should be used with caution. This example is intended to highlight that there is likely NEC in the disordered regions. What the structure of this lattice may be is not clear.

**Fig. 2:**
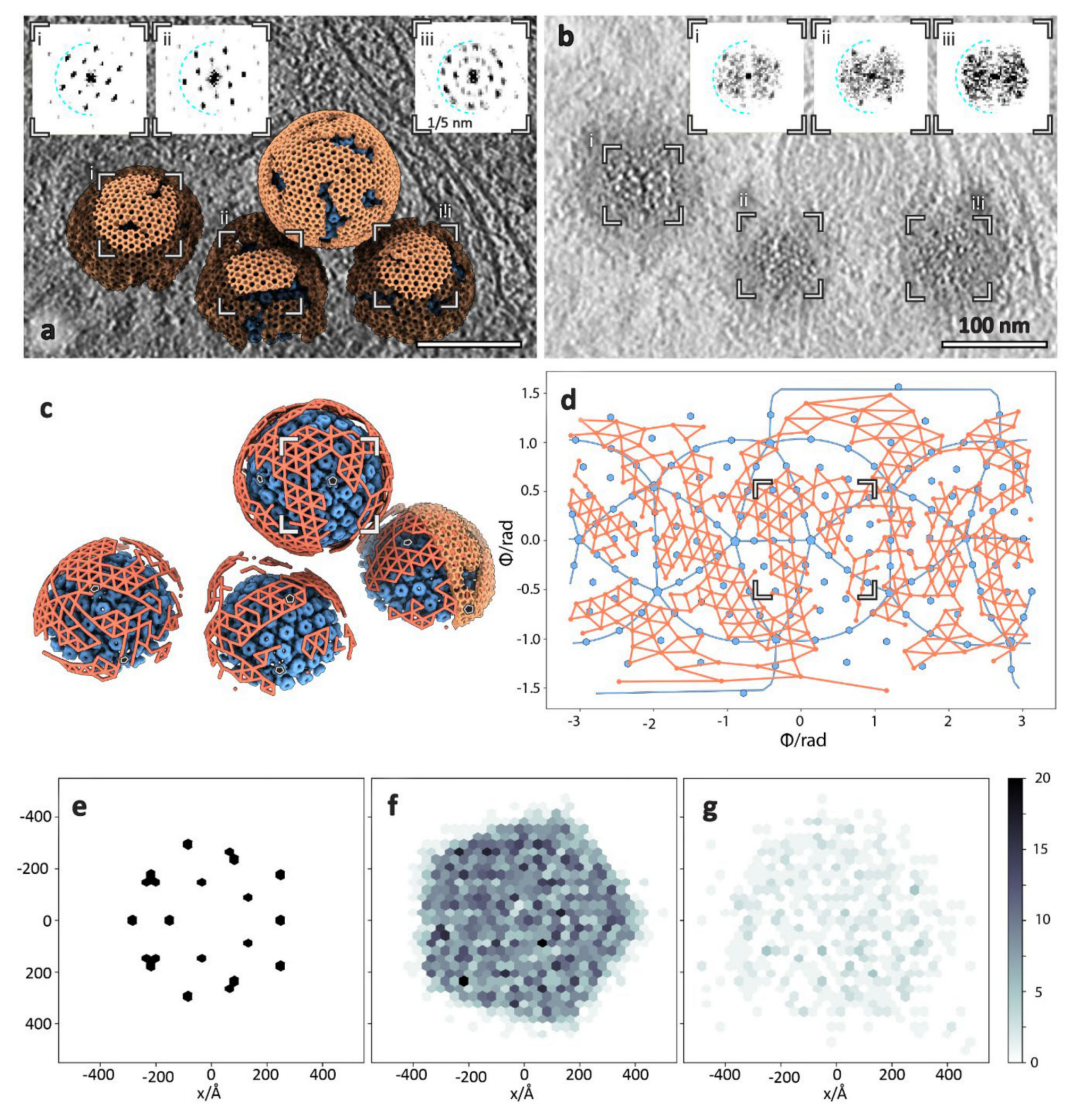
PrV NEC forms a locally ordered lattice. **a**, A three-dimensional rendering of the NEC and nucleocapsids generated by backplotting subvolume averaging maps combined with a transparent tomogram slice. The insets show the contrast adjusted power spectra of sections through the highlighted areas intended for comparison to the raw data shown in **b**. The hexagonal arrangement of peaks in (**i**) and (**ii**) indicates a well ordered hexagonal lattice, while (**iii**) indicates the presence of multiple lattice orientations and by extension a disordered transition between them. Scale bar, 100 nm. **b**, A section through the same tomogram showing tangential views of three perinuclear NEC vesicles and their respective power spectra (compare with **a**; the opacity around the highlighted area has been reduced). **c**, The same particles, but with the NEC lattice represented schematically by lines connecting hexamer centres. Some capsid vertices have been indicated with pentamers to indicate their orientation. **d**, Polar coordinate representation of **c**, with the origin at the nucleocapsid centre. Blue lines represent icosahedral edges. **e, f, g**, Histogram of the relative distribution of hexons (**e** used as a control), perinuclear NEC hexamers (**f**) and budding NEC hexamers (**g**) relative to penton vertices. Scale bar represents the number of particles in each bin. As expected, hexon positions show a regular arrangement relative to the fivefold vertex, whereas the NEC positions appear to be randomly distributed.

The majority of perinuclear vesicles contained nucleocapsids (182 out of 229), which is a clear indication that there is a specific NEC-nucleocapsid interaction that triggers perinuclear vesicle formation, if not also the initial assembly of pUL31/34 into a curvature-inducing lattice. As both NEC and nucleocapsids are periodic structures, their specific interaction could manifest as an alignment between their respective geometries. We determined the structures of the NEC hexamer and capsid pentons and hexons in perinuclear vesicles using subvolume averaging (Methods). This allowed us to determine their structures, and accurately and empirically located their positions, enabling an analysis of their correlation. To verify that these measurements accurately represent the lattice geometry, we placed NEC hexamers back into the original volume (backplotted) and compared the resulting model to the original data; the two were a good match both in real and reciprocal space (Fig. 2a, b). The hexameric NEC formed ordered domains across the surface of the vesicles, with patches of disorder evident in between areas where the hexagonal symmetry persisted (Fig. 2c, d, Extended Data Fig. 4). However, we noticed no discernible pattern in the alignment of the NEC and icosahedral lattices in individual perinuclear vesicles (Fig. 2c, d), or by global analysis of the distribution of NEC hexamers relative to capsid pentons (Fig. 2e, f).

**Extended Data Fig. 5:**
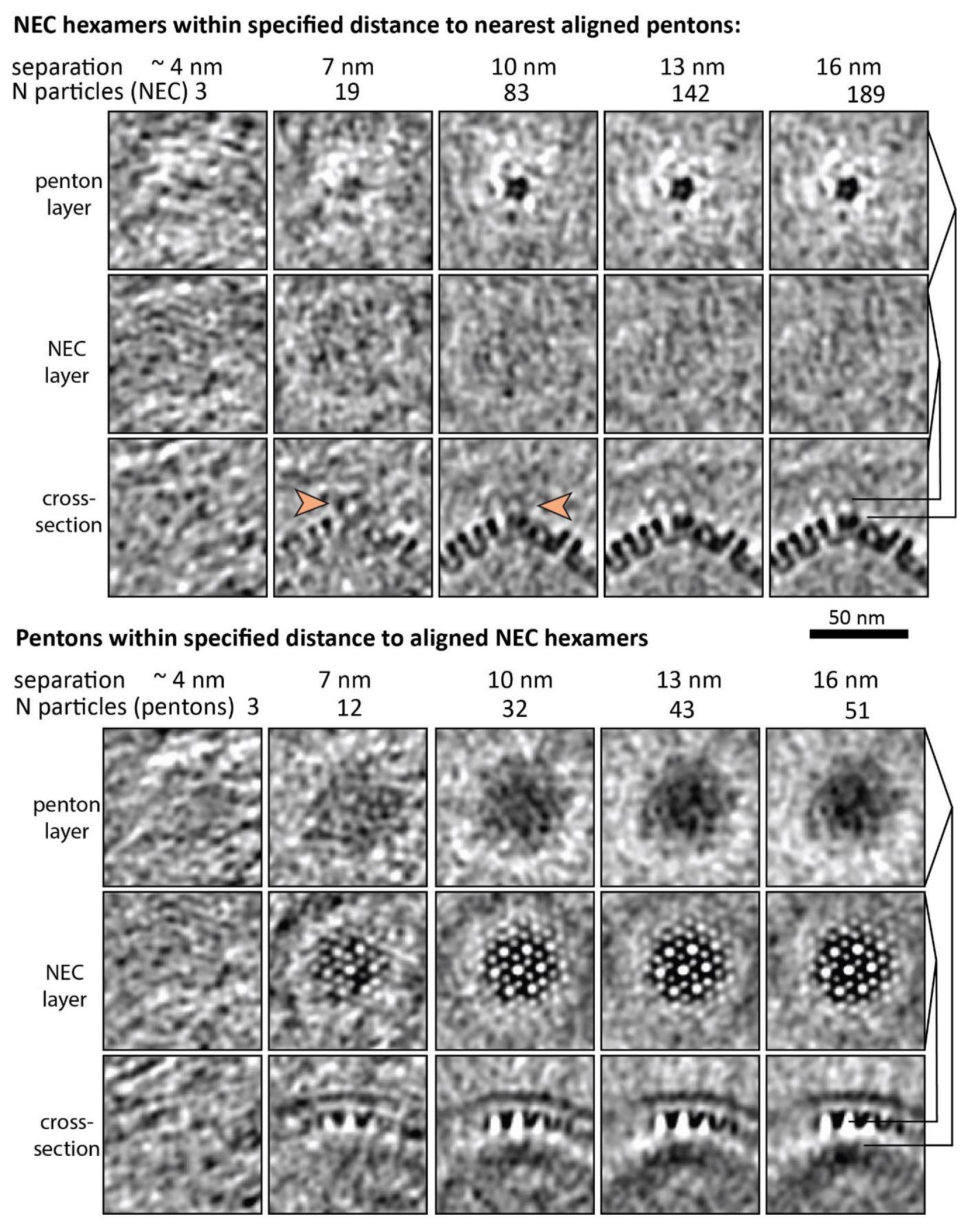
Questionable connecting densities between nuclear capsids and budding NEC. The NEC subvolumes were classified by the distance, to the nearest penton vertex (top panel) and vice versa (bottom panel). Each column shows sections through the resulting class average volume, with the maximum separation distance and the number of particles included in the average indicated above. All volumes were filtered using the same bandpass filter. The NEC layer is smeared in the penton-aligned averages and accordingly the capsids are smeared in the NEC-aligned averages, indicating that the two lattices are not aligned. There is a hint of a density originating from pentons closer than ~7 nm from the nearest NEC surface (orange arrows), but any interpretation of this would be highly questionable due to the small number of particles.

**Fig. 3:**
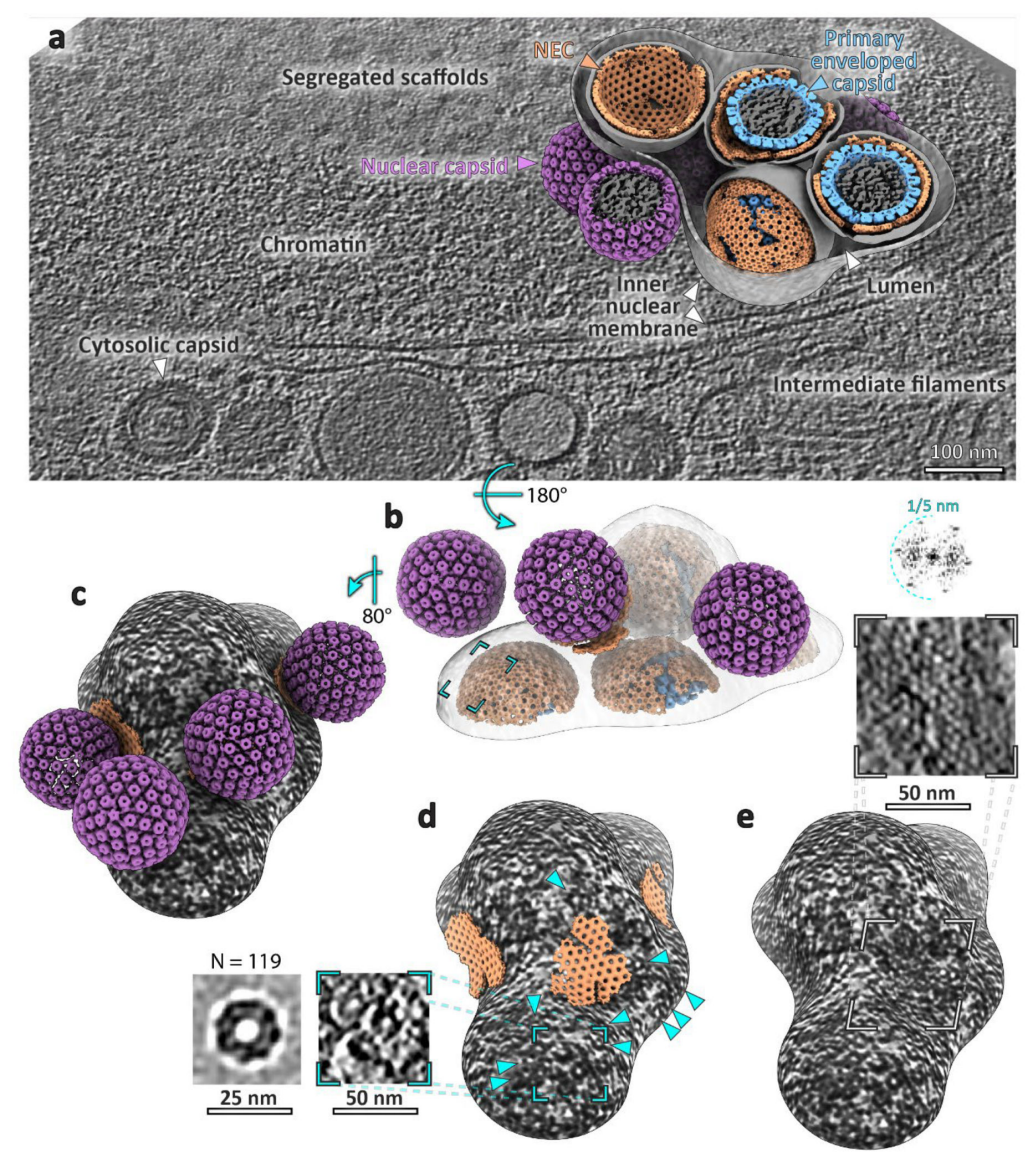
Initial stages of primary envelopment. **a**, A slice through a tomogram and a backplotted three-dimensional representation of egress components. The membrane of one NEC vesicle has been cut away to expose its contents; similarly, one nucleocapsid volume has been omitted to show the inner NEC surface. INM, inner nuclear membrane. **b-e**, The same rendering, but flipped upside down to show the detail of assembling NEC and capsid interaction. **b**, The INM is shown at low opacity and perinuclear vesicle membranes are not shown. The cyan box marks the same position as in **d. d**, The arrowheads indicate putative ring-like NEC located on a negatively curved (concave) membrane (insets show slices through an average volume of 119 particles and a through the tomogram at the highlighted position, Extended Data Fig. 6). **e**, Insets show a slice through the raw tomogram and the power spectrum of the highlighted positive curvature area.

Aiming to determine whether there is a specific capsid-NEC interaction leading to the initiation of primary envelopment, we focused our attention on nuclear capsids in the proximity to the INM. In the majority of cases (19 out of 28, counted in the 8 highest quality tomograms) where nuclear capsids came within 20 nm of the INM, we observed patches of NEC already assembled in a hexameric array, as evidenced by the sixfold peaks in their power spectra (Fig. 3d). These patches had a positive curvature, similar to fully formed perinuclear vesicles. Assessing these data globally (Fig. 2g) and on an individual case basis (Supplementary Figs. 1, 2) again showed no correlation between the respective symmetries. To further interrogate our dataset for hints of capsid-NEC contacts that could be mediated by pUL17/pUL25, we classified our dataset of putative budding events based on the penton-NEC distance. Having obtained accurate positions and orientations of nucleocapsid pentons, we extracted and averaged the volumes of nearby NEC and vice versa (Extended Data Fig. 5). The resulting densities can be interpreted similarly to the coordinate histogram representation (Fig. 2e-g) with the important caveat being that the density does not depend on specifying reference coordinates relative to either lattice. Again, there was no evidence of pentons being aligned with any part of the NEC lattice. Weak bridging densities originating at penton vertices at ~7 nm separation could be observed, but these are weak and may be an artefact of averaging low numbers of particles (Extended Data Fig. 5).

**Extended Data Fig. 6:**
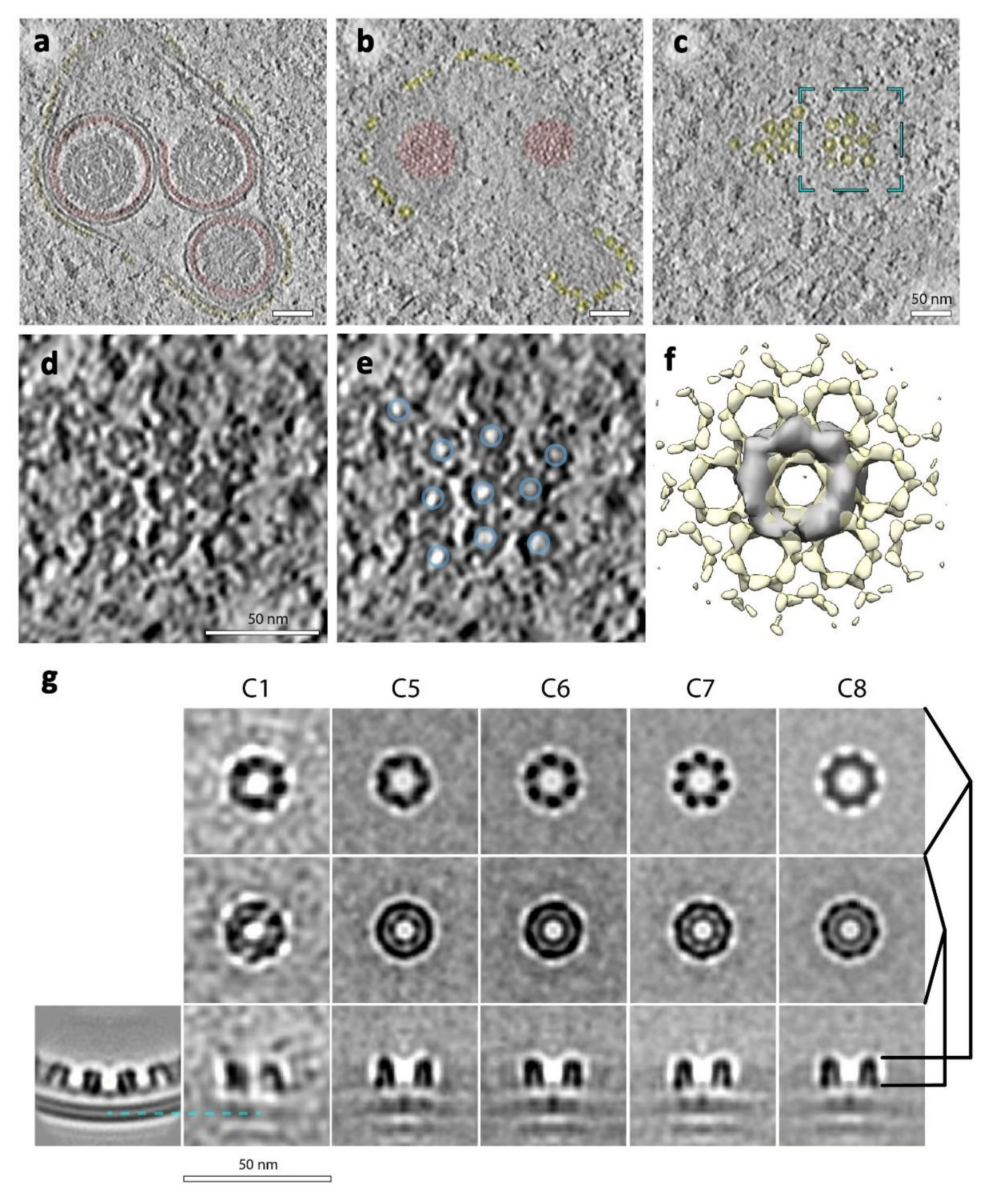
Putative NEC coat on negatively curved surfaces. **a, b, c**, Slices through the same nucleoplasmic reticulum at different depths showing the distribution of the putative NEC layer. **d, e**, An enlarged section of panel c, showing top views of ring-like structures (highlighted in blue in e). **f** Overlay of the surface representation of the average volume of 121 ring-like particles from two tomograms and the spherical NEC lattice. Each ring could plausibly accommodate two concentric layers of pUL31/34 dimers. Averaging a more exhaustive (but less stringently picked) set of negatively curved lattice particles did not converge (and is therefore not shown), suggesting a high degree of variability. Notably, the membrane was not included as an alignment feature. **g**, The thickness and distance to the membrane of this layer are consistent with the spherical NEC lattice. Sections through the ring average volume with different C symmetries applied. Visually, C7 is the best match to C1 but it is possible these structures have no strict symmetry, as suggested by d, e. Note that symmetrisation in this case means addition of subvolumes at defined rotations (for example 5 subvolumes with 60° degree increments for C6 symmetry). Alignment was performed after the addition of symmetry related particles. The bottom left-most panel is a section through the spherical NEC lattice.

Interestingly, while inspecting the nascent budding sites, we noticed an electron dense coat, both on flat and negatively curved INM surfaces with cross-sectional dimensions similar to those of positively curved NEC (Fig. 3d, Extended Data Fig. 6). This layer consisted of a pseudo-lattice of irregular rings approximately 25 nm in diameter. These rings could be seen surrounding areas of regular lattice, suggesting that they are either a precursor or an intermediate form of the NEC. Subvolume averaging of these rings did not result in a density map with a clear rotational symmetry, hinting at extensive heterogeneity and flexibility (Extended Data Fig. 6g).

### A fully assembled CVSC first appears in the cytosol

**Fig. 4:**
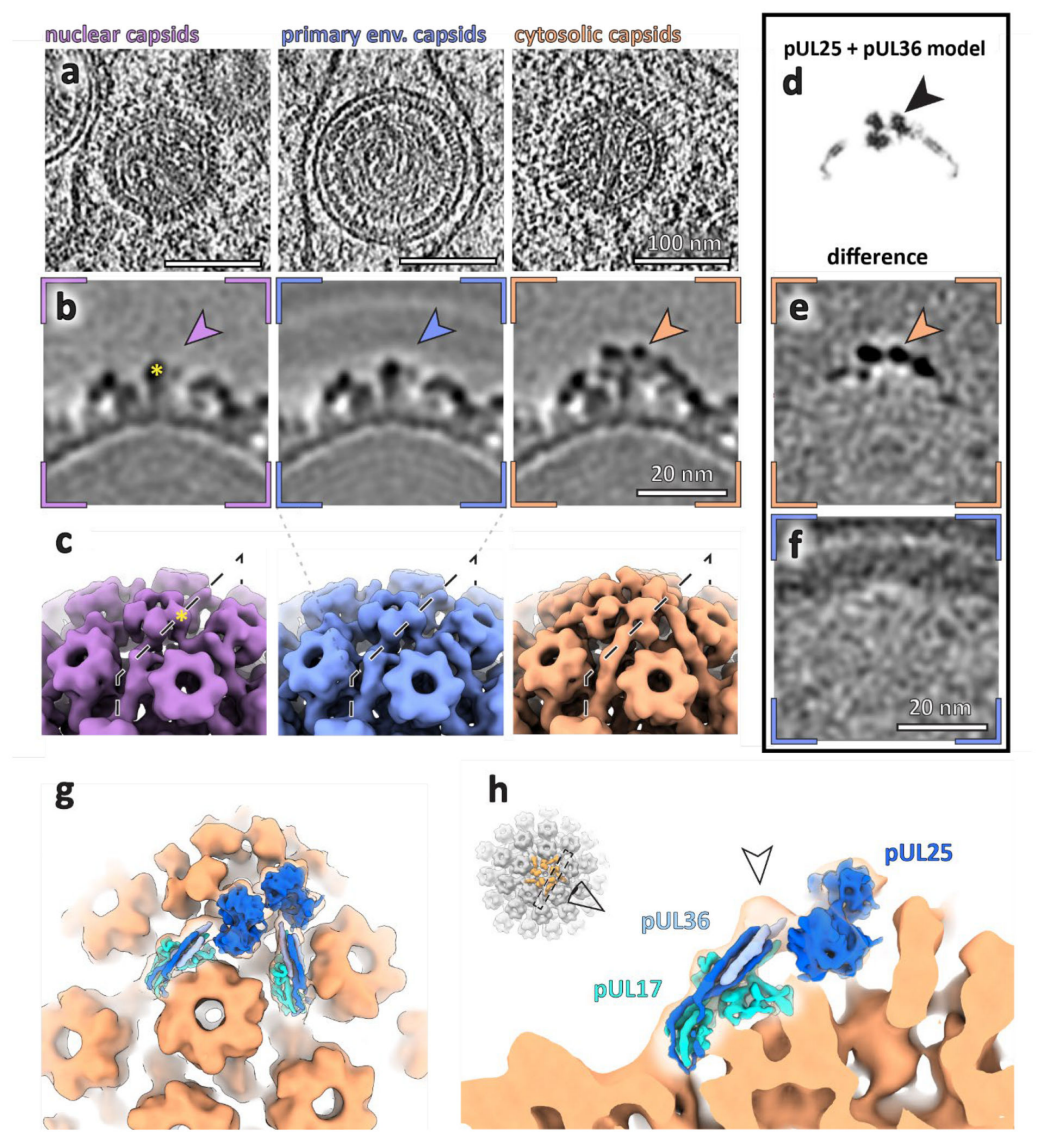
Subvolume averaging of PrV capsids in different subcellular locations reveals the compartment-dependent assembly of penton vertex components. **a**, Raw tomographic slices of C-capsids within specified locations within the cell. Scale bars, 100 nm. **b**, Slices through the respective average volumes. The arrowheads indicate the position of densities present only in cytosolic capsids. The scale bar applies to all images in **b**. Yellow asterisk in **b** and **c** mark the same penton domain. **c**, Surface representation of volumes shown in **b. d**, Slice through a simulated density map of two capsid vertex specific component (CVSC) proteins pUL25 and pUL36 (PDB 2F5U^34^, 7FJ1). Images in **d-f** are shown at the same scale. **e**, Normalised difference map between nuclear and cytosolic capsids. **f**, Normalised difference map between nuclear and perinuclear capsids. **g, h**, Simulated density of CVSC components (PDB 2FSU, 7FJ1) fitted into cytosolic capsid map (orange). **h**, The arrowhead indicates a density unaccounted for by existing atomic models (but present at low contour levels in for example, EMD-6387 and EMD-31611).

It has been proposed that the CVSC, especially pUL17/25 (among others^16,32^), is responsible for mediating the initial capsid-NEC interaction. Failing to determine any link between NEC symmetry and the capsid icosahedral orientation, we therefore investigated whether there were any structures on the capsid that may indicate any difference in protein complement between perinuclear vesicles and nuclear or cytosolic capsids. We therefore obtained structures of the icosahedral vertices (pentons) to probe the molecular structure at this potential interaction site from PrV capsids contained within the nucleus, perinuclear vesicles, and the cytosol (Fig. 4, Supplementary Figs. 3, 4). This enabled an assignment of individual capsid proteins on the penton and a comparison of their molecular arrangement during nuclear egress.

The nucleocapsids contained within the nucleus showed electron microscopy densities for pUL17, which forms the base of the CVSC in fully-formed virions and was suggested to be present in different numbers dependent on capsid type (A-, B- and C-type capsids^1^); averaging the different types of capsids from within the nucleus did not reveal major differences in structure at the resolutions we were able to achieve (Supplementary Figs. 3, 4, Supplementary Table 1). Similarly, there was no significant difference between nuclear capsids and those determined within perinuclear vesicles (24 Å, Fig. 4f). Consistent with our analysis of budding events, the NEC layer was smeared with no obvious densities connecting the NEC layer and pUL17.

In contrast to nuclear and perinuclear capsids, A-, B-, and C- capsids in the cytosol had a clear density for the full CVSC, previously shown to be present in fully formed HSV-1 and PrV capsids^19,33^. The combined average of cytosolic capsids at ~20 Å resolution showed a good fit for both pUL17 and pUL25 (Fig. 4g, h). The dimensions of the density were consistent with the presence of the N-terminal portion of pUL36 previously seen in high-resolution structures of virions^1^. A difference map between the nuclear and primary particle capsid structures highlighted that the 6-helix bundle (pUL17, pUL25 and pUL36 helices) and the pUL25 globular domains are the major structural differences between capsids from the different subcellular regions (Fig 4d, e).

**Extended Data Fig. 7:**
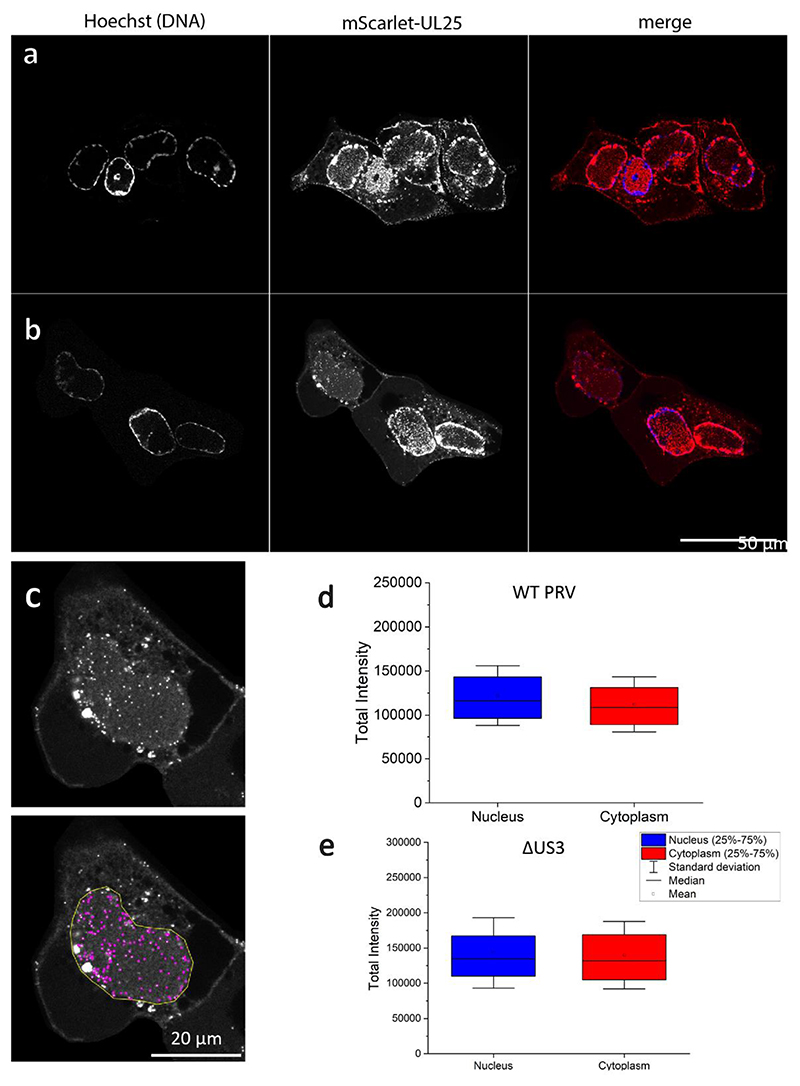
Localization of individual nuclear and cytoplasmic mScarlet-UL25 labelled capsids. **a**, PK15 cells were infected with PrV-mScarlet-UL25 or **b**, PrV-mScarlet-UL25-ΔUS3 fixed at 7 or 10 hpi, respectively, and imaged using spinning disc microscopy. UL25-mScarlet (red); DNA-Hoechst (blue). One plane of the acquired volume is shown. **c**, Viral particles were detected in the 3D volumes using Trackmate in FIJI with an expected blob diameter of 0.4 microns and the quality threshold set to 10.0. **c**, Fluorescent signal in a single plane of a PK15 cell infected with PrV-mScarlet-UL25-ΔUS3 and fixed 10 hpi (top) and projection of all detected single particles (purple) of the volume in a nuclear ROI (yellow) onto one plane (bottom). **d, e**, The FIJI plugin Trackmate was used to detect and measure individual virus particle fluorescent intensities. For each condition, the total intensity of more than 4,000 particles was quantified and detected in more than 30 different cells, all using a single biological replicate.

pUS3 has been shown to be present on PrV capsids^35^, however, it is unclear that the deletion of pUS3 impacts upon the ability of pUL25 to associate with capsids in situ. Therefore, we attempted to determine whether the level of recruitment of pUL25 in the nucleus is affected by deletion of pUS3. First, we verified that pUL25 is present at the same levels in WT PrV and the ΔUS3 mutant using fluorescence microscopy (Extended Data Fig. 7), showing that the recruitment of pUL25 is unaffected by US3 deletion. We then verified the CVSC assembly patterns using FIB-SEM and cryoET on WT PrV infected cells. As egress proceeds rapidly in the case of WT PrV, we observed no perinuclear vesicles, but the patterns of nuclear and cytosolic CVSC recruitment were identical to the ΔUS3 mutant. Similarly, procapsid assembly occurred in clusters or kindergartens despite the late stage of infection^36^ (Supplementary Fig. 5). Therefore, using PrV-ΔUS3 system reliably represents the processes that take place during WT PrV infection. The CVSC assembles once capsids enter the cytosol and it is not required for egress.

### Unexpected lattice flexibility of the NEC

**Fig. 5:**
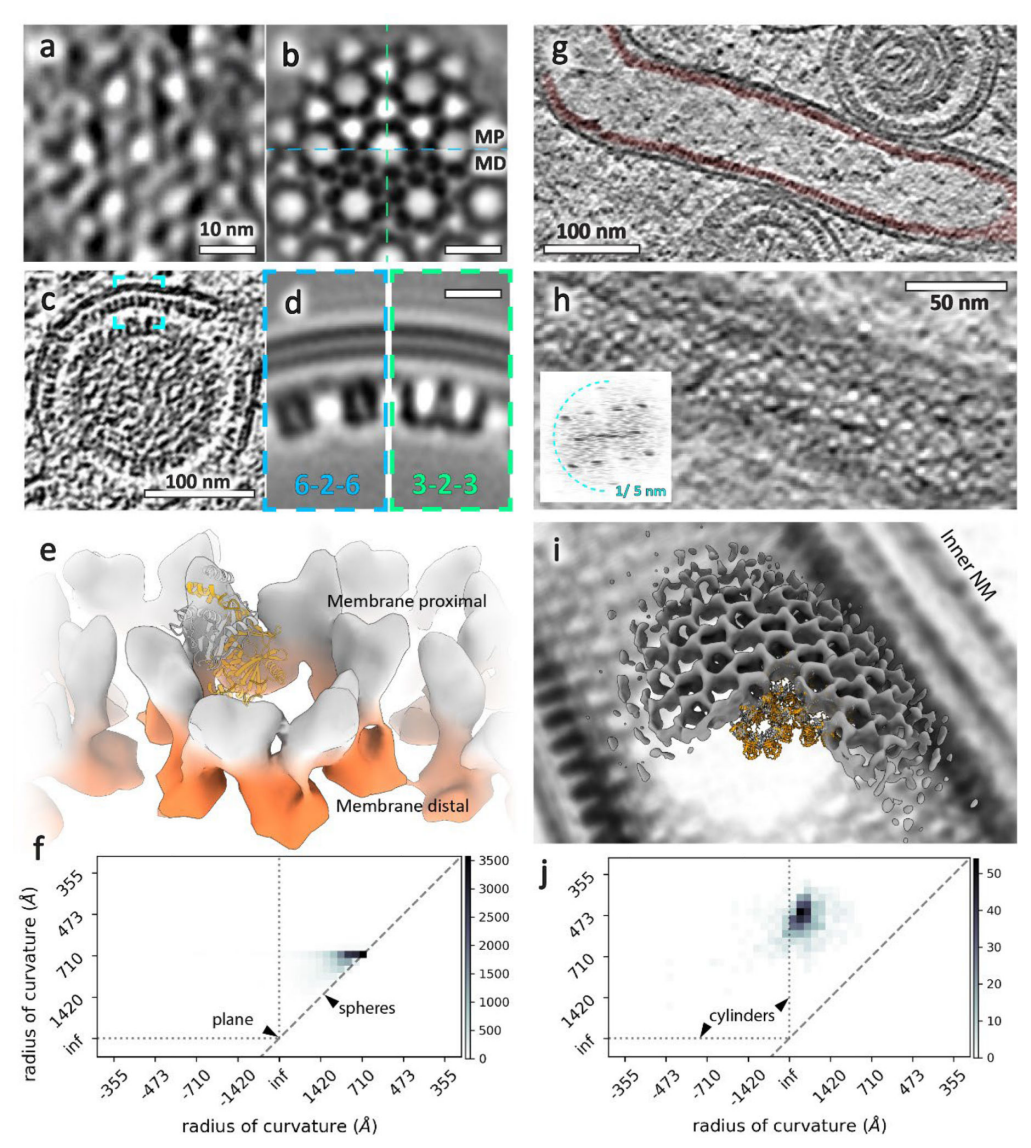
Subvolume averaging of the PrV NEC. **a-e**, Spherical NEC; **g-i**, tubular (helical) NEC. **a**, Tangential section through the NEC showing a lattice top view. **b**, Tangential sections through the NEC average volume at the membrane proximal (MP, top) and membrane distal (MD, bottom) layer. **c**, Equatorial section through a perinuclear vesicle showing the NEC lattice side-views. **d**, Sections along the 6-2-6 and 3-2-3 lattice directions (also indicated in **b**). **e**, Isosurface representation of the average volume with the membrane proximal and distal layers highlighted in grey and orange, respectively (also shown in Supplementary Fig. 6). **f, j**, Curvature analysis: two dimensional histograms of radii of curvature of NEC particles from spherical vesicles (f) and tubular vesicles (j). The diagonal dashed line in each represents spherical curvature. The vertical and horizontal dotted lines represent an infinite (inf) radius of curvature in one direction, thus describing a cylindrical surface. In this case, a negative radius of curvature represents a concave NEC surface (that is, with the NEC on the outside of a spherical vesicle). The histogram peak for the tubular NEC is ~40 nm and for the spherical NEC it is ~64 nm. The remaining spherical particles tend toward ellipsoidal curvature rather than a sphere. Scale bars indicate the number of particles (NEC hexamers). **g**, Slice through a tomogram showing a tubular NEC form (red) in type-1 nucleoplasmic reticulum. **h**, Top view of the same tube showing a regular lattice arrangement. The inset shows the power spectrum of the projection through the lattice. **i**, Isosurface representation of an average volume generated from four NEC tubes (overlaid with a density representation of a single tube). INM, inner nuclear membrane.

As mentioned previously, we used subvolume averaging to determine an electron microscopy density map of the NEC coat. The aim was to analyse the molecular architecture of the NEC and to further probe whether any intermolecular interactions with capsids could be elucidated, or if we could determine the molecular basis for curvature in these vesicles. We averaged all NEC-containing vesicles irrespective of size or shape. The final average reconstructed from ~135,000 particles reached ~ 14 Å resolution and shows a good match to previous data reconstructed from empty pUL31/34-GFP vesicles^10^ (Fig. 5, Supplementary Fig. 6).

**Extended Data Fig. 8:**
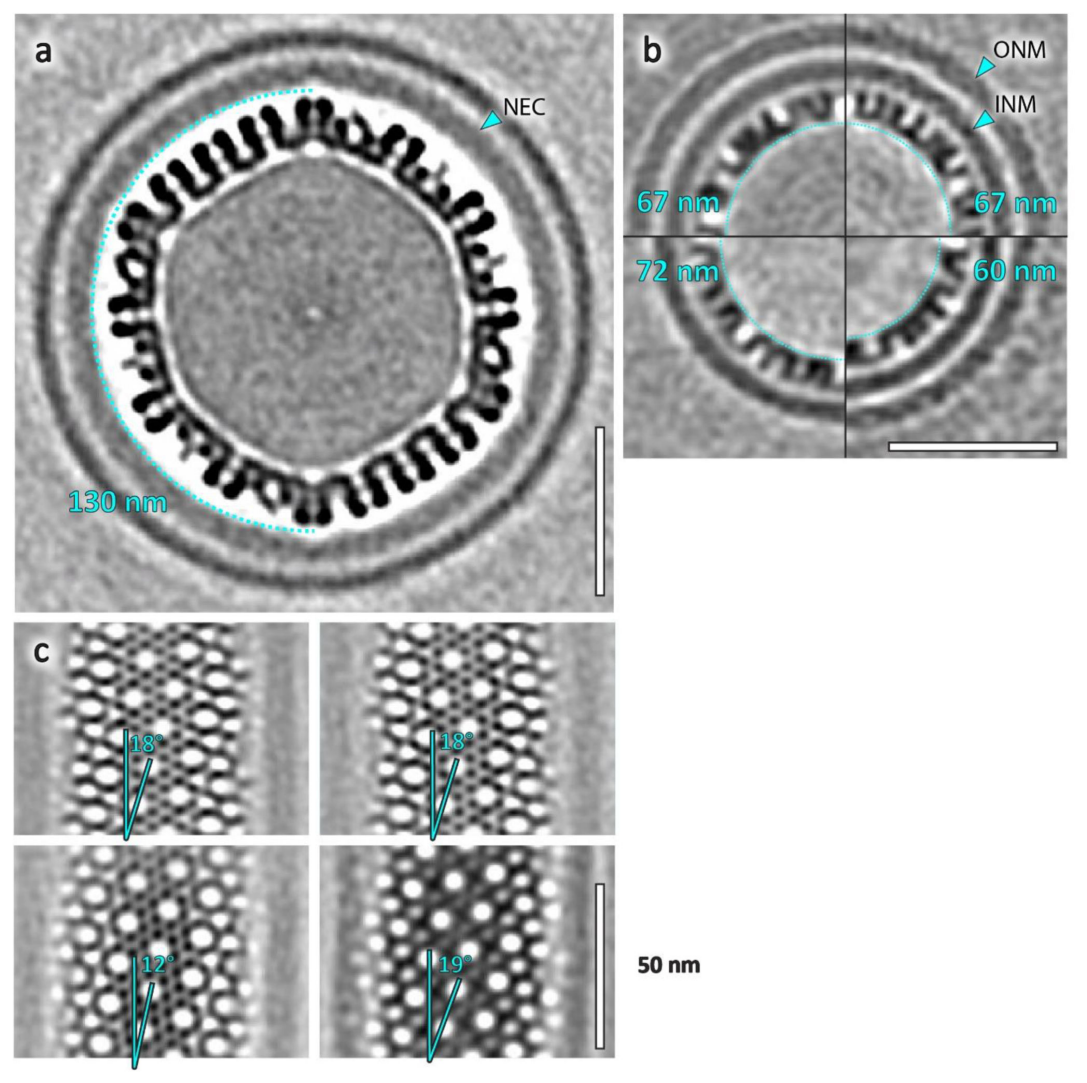
PrV nuclear egress complex forms tubes with helical symmetry. **a, b**, Comparison of the NEC curvature in perinuclear vesicles and tubes. **b**, Shown are sections through the average volumes of four tubes, with the respective diameters indicated in cyan. The NEC tubes have substantially smaller luminal cavities compared to spherical NEC vesicles. Notably, the two separate tubes with matching diameters and helical parameters were located within the same nucleoplasmic reticulum and may have originated from the same assembly. **c**, Tubular NEC forms tubes with different helical parameters, indicating a flexibility in the direction of largest curvature. Highlighted is the angle of the 6-2-6 axis to the helical symmetry axis. Scale bars indicate 50 nm.

Furthermore, we determined the structure of the NEC in perinuclear tubes. The lattice on the inside of tubes was well ordered relative to perinuclear vesicles, resulting in a ~21 Å resolution map despite a limited number of particles (Fig. 5i). Small deformations did not correlate with a loss of long distance helical order, however, there were clear breaks in the lattice around bends (Fig. 5g). The inner diameter of observed tubes was between 60 nm and 72 nm, nearly half the diameter of the spherical perinuclear vesicles and even empty vesicles reported previously^10^ (Extended Data Figs. 2, 8). Notably, we were able to determine the structures of individual tubes with three different diameters and therefore three different helical parameters.

There was a third type of hexameric lattice in our data, a relatively flat double-layered sheet bringing together two surfaces of the INM (Fig. 1g, Extended Data Fig. 3). Interestingly, while the dimensions of individual hexamer subunits match those of spherical NEC, the spacing between hexamers is ~2 nm larger in the flat lattice (Extended Data Fig. 3g, h).

### Inter-hexamer spacing is responsible for curvature induction

**Fig. 6:**
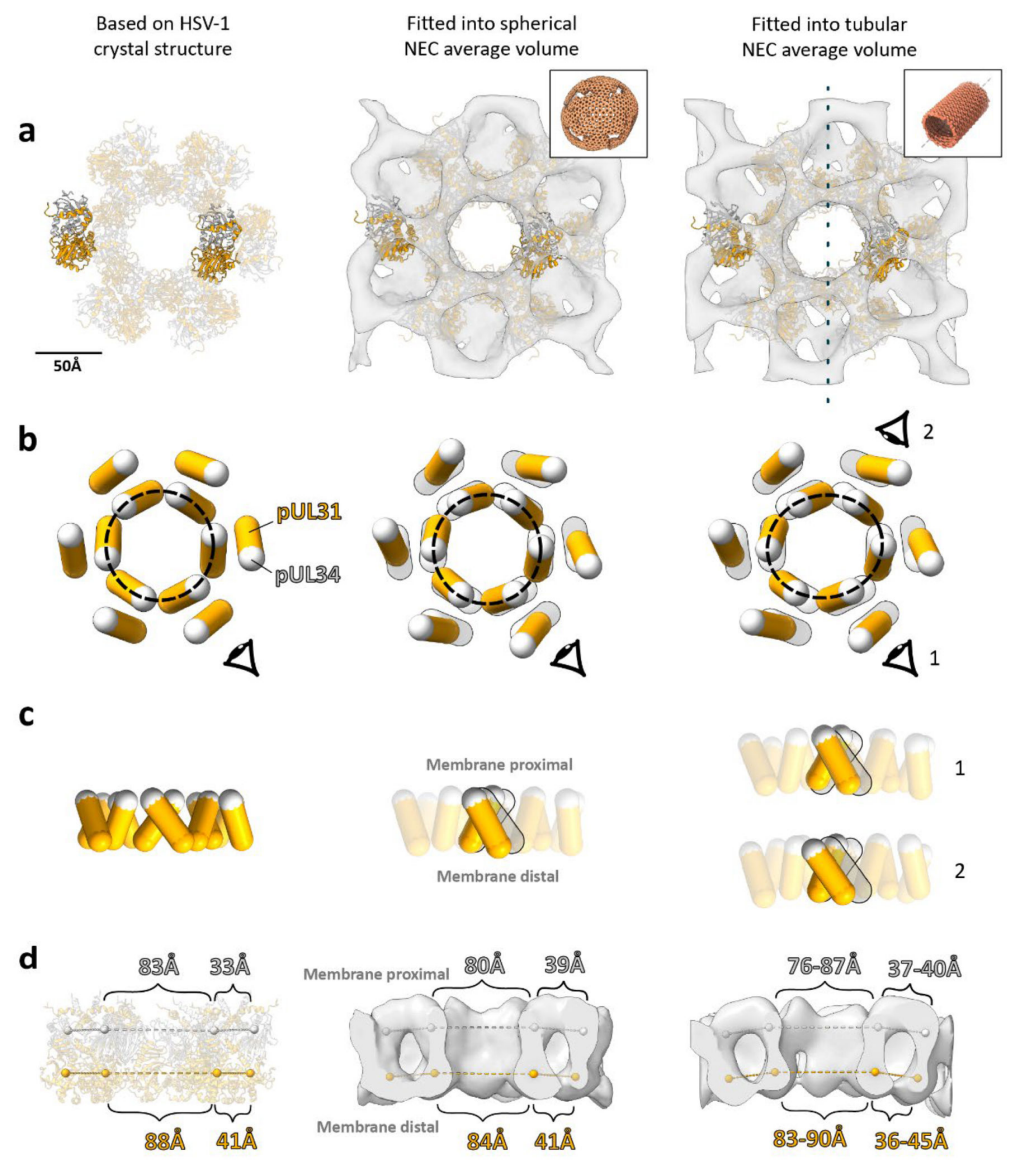
A comparison of different PrV NEC lattice structures, created by fitting PDB 4Z3U chains C/D into various structures. Left column, hexamer created by superposing PrV NEC (PDB 4Z3U) into heterodimer positions from crystal symmetry positions in HSV-1 NEC (PDB 4ZXS). Middle column, heterodimer positions found by rigid body fitting of 4Z3U into the subvolume average of the NEC from spherical/non-tubular vesicles. Right column, heterodimer positions found by rigid body fitting into the subvolume average of NEC from tubular regions. **a**, View of the three lattices with membrane proximal regions facing the viewer. Subvolume averages are shown as a grey isosurface, pUL31 as an orange ribbon, and pUL34 as a light grey ribbon. The dotted line in the tubular average (right) indicates the orientation of the helical symmetry axis. The insets depict the overall lattice architecture from which the averages have been made. **b**, Lozenge representation of heterodimers as seen in row **a**. Light grey markers represent centre of mass of pUL34 and arm of pUL31 (residues 18-55); orange markers represent the centre of mass of the remainder of pUL31. Shadow lozenges in the middle and right columns represent the crystal structure-based model. The dotted grey ellipses show the shape of the central hexamer, as described by the centres of mass of pUL34 and the arm of pUL31. **c**, Side view of lozenges as represented in **b. d**, Distances between centre of mass markers on opposing sides of the NEC hexamers. The light grey and orange markers are as described in **b**. The tubular hexamer in the right column was elliptical, and hence the minimum and maximum distances are shown.

At the resolution of our subvolume average of the NEC lattice, a detailed all-atom analysis of conformational changes or interactions between and within individual heterodimers is not possible. However, attempting to fit the crystal structure of PrV pUL31/34 (PDB 4Z3U) into the map does generate some broad insights into the native, curved NEC structure. A simple rigid fit of multiple heterodimers shows that the lattice suggested by our map is consistent with the hexameric crystal lattice of the HSV-1 NEC^37^ (Fig. 6A); despite the obvious differences in long range curvature, the two lattices bear strong resemblance to one another. A comparison between the PrV pseudoatomic model and the HSV-1 crystal structure showed the largest differences to be in the inter-hexamer spacing (Fig. 6d, left and middle images). The membrane distal inter-hexamer distances are roughly the same, but the membrane proximal distances are larger in the PrV lattice by ~6 Å. Notions that the PrV NEC hexamer remains a relatively inert unit, however, are broken by similar analysis of the heterodimers in the helical NEC average (Fig. 6, right column). Here, the intra-hexamer distances vary substantially, both from the previously analysed structures, and from themselves. The hexamer is stretched to form an ellipse, with the major axes 11 Å larger than the minor axes at the membrane proximal region, and 7 Å larger at the membrane distal region.

### Common features in nuclear egress of WT PrV and WT HSV

**Extended Data Fig. 9:**
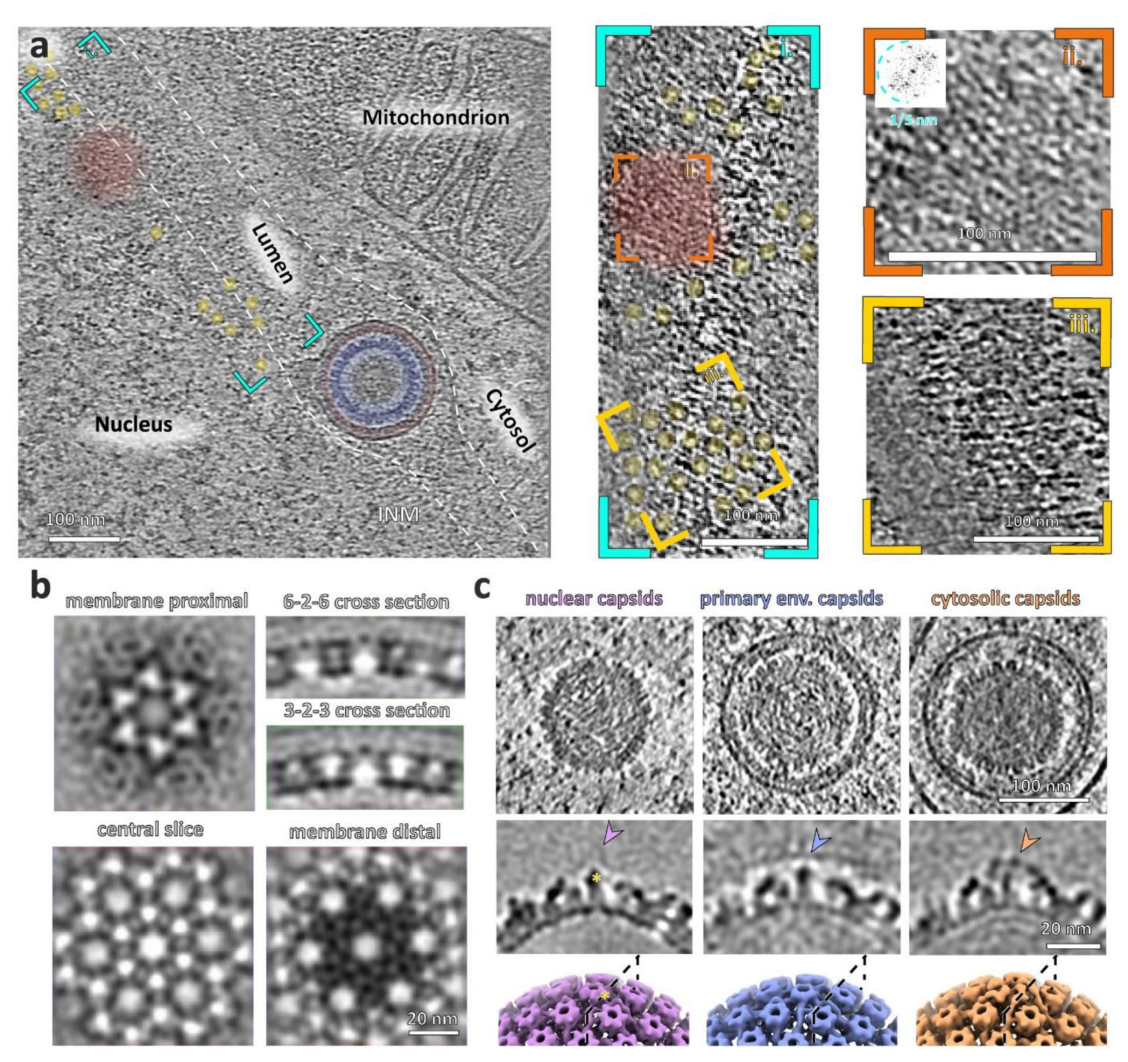
NEC in HSV1. **a**, Slice through a tomogram depicting primary enveloped nucleocapsid egressing to the cytosol. The NEC coat of WT HSV-1 (red) follows the same pattern as that of ΔUS3 PrV and could be identified on the INM and in perinuclear vesicles. Likewise ring-like structures similar to those in PrV (yellow in i. and iii., Fig. 3c and Extended Data Fig. 6) was identified near the hexagonal NEC lattice (red in i. and iii.). Red area in the inset ii. shows budding NEC. **b**, HSV1 NEC crystal structure (PDB 4ZXS) fitted into the subvolume average of HSV-1 NEC from perinuclear vesicles. ONM, outer nuclear membrane; INM, inner nuclear membrane. **c**, Raw tomographic slices through C-capsids of WT HSV-1 in indicated subcellular locations. **d**, Slices through the average volumes of C-capsids (middle) and their surface representations (bottom). Arrowheads indicate the position of an additional density present in the cytosolic capsids. 15 tomograms from 5 preparations were used.

Seeking to determine whether the patterns of nuclear egress we established are unique for PrV or are more broadly applicable to *Alphaherpesvirinae*, we performed FIB-SEM and cryoET on HSV-1 infected cells (Extended Data Fig. 9). Consistent with the high rate of egress of WT HSV-1 in its natural host, we observed no egress events in HFF cells. There were, however, ring-like NEC structures present on the INM resembling those found in the ΔUS3 PrV system (compare with Extended Data Figs. 3, 9). Using Vero (African green monkey) cells, we were able to capture four perinuclear vesicles and one event of NEC budding (Extended Data Fig. 9a), and could therefore confirm that the CVSC is only fully assembled (or ordered) in the cytosol (Extended Data Fig. 9c). Subvolume averaging of ~9,000 particles of the HSV-1 NEC from perinuclear vesicles resulted in a map with ~27 Å resolution (Supplementary Data Fig. 7). The map is similar to that of the PrV NEC, with subtle differences. However, given the poor fit of the HSV-1 NEC crystal structure, probably resulting from the small amount of data of this rare process, we chose not to interpret these differences on the molecular level.

## Discussion

The data presented here show that the NEC forms a large number of oligomeric structures capable of modulating membranes both in the presence and in the absence of nucleocapsids. While the NEC can bud into the perinuclear space in the absence of other viral genes^10^, the majority of perinuclear vesicles contained capsids or capsid assembly intermediates (Extended Data Fig. 2c). This indicates that a specific molecular interaction takes place between capsids and the NEC. Multiple studies have suggested that the pUL25/pUL17 components of the CVSC are needed to trigger egress. However, here we show that the full CVSC assembles only after capsids reach the cytosol (Fig. 4), despite pUL25 being apparently present on nuclear capsids at a similar occupancy to cytosolic capsids (Extended Data Fig. 7d, e) ^38^. We found no structural evidence for any single capsid component making preferential contact with the NEC (Fig. 2, Supplementary Figs. 1, 2 Extended Data Fig. 5), suggesting that it is transient or mediated by flexible components. Our observations lead to the conclusion that NEC-capsid recruitment is not a rigid process, with capsid proximity not necessarily being a driver of NEC formation and elongation.

As it is impossible to tile a sphere with hexagonal units, we hypothesise that the NEC forms spherical vesicles through hexagonal sheets with short-range spherical curvature separated by areas of disorder, analogous to the immature Gag lattice in HIV-1 virions^39,40^. Consistent with our hypothesis, we observed elongated hexagonal patches more than ~120 nm long but only up to ~60 nm wide, whose local curvature is nevertheless chiefly spherical with a limited tendency towards tubular curvature (Fig. 5f, j). Interestingly, there are NEC-like lattices, albeit irregular, in between hexagonally arrayed areas (Fig. 2b, Extended Data Fig. 4). This suggests that the NEC can form relatively stable contacts outside its preferred oligomeric state and could explain why an NEC pentamer could form on a penton vertex scaffold in vitro^23^. This flexibility is probably required to compensate for the deformations caused by spherical curvature applied to a hexameric lattice, but also explains the ability of the NEC to form vesicles of varying morphologies. The magnitude of induced membrane curvature is determined by the cargo, however, this is not mediated specifically by proteins at the fivefold vertex of the capsid, but rather by complementary electrostatic interactions^41^. These data are consistent with previous observations that herpesvirus egress is similar to ribonucleoprotein aggregate removal from the nucleus of *Drosophila* cells^10,42^, insofar as pUL34/31 heterodimers can associate to form intraluminal arrangements diverse in both size and geometry.

The diversity of lattice geometries presented an opportunity to study the conformational changes involved in curvature generation in situ and without perturbations by mutagenesis. Firstly, we show that on a short scale, the native PrV NEC lattice is consistent with the HSV-1 crystal lattice, suggesting that the model from a previous study, made using a map with much lower resolution and supported by analysis of point mutant functionality, is inaccurate^41^. The comparison of pseudoatomic models generated from spherical and tubular vesicles indicates that curvature in spherical vesicles arises from changes in the inter-hexamer distance, an observation that could not be made in a rigid crystal lattice. The hexameric unit stays relatively intact, while the pUL34 portions between hexamers move further apart compared with pUL31. Inducing curvature past a certain point may require an asymmetric deformation of the hexameric unit; this would be consistent with the helical NEC lattice adopting tighter curvature than even empty spherical vesicles.

The NEC plasticity is further demonstrated by two non-canonical structures, a putative precursor NEC pseudo-lattice consisting of irregular rings and a double-layered flat hexagonal lattice (Figs. 2g, 3d, Extended Data Figs. 3, 6). Interestingly, both a putative double-layer NEC and individual rings have previously been observed on negatively curved surfaces in vitro^22^. Considering that the membrane distal part of pUL31 is negatively charged, this configuration must require a conformational change that allows homotypic interaction with complementary electrostatics. This may explain the difference in subvolume average structures between these NEC lattices, such as the hexamer-hexamer distance being substantially larger in the double-layer NEC compared with the spherical and helical lattices (Extended Data Fig. 3j). Together, these structures allow an insight into the flexibility of NEC heterodimers and further showcase the range of structures that the pUL31/pUL34 heterodimer can adopt. Further work will be needed to understand whether there is a role for these arrangements in the herpesvirus life cycle or if they are an evolutionary redundant pathway relating to molecular remodelling of nuclear membranes.

## Materials and Methods

### Cell Culture

#### PrV-ΔUS3

A mutant PrV-ΔUS3 virus^43^ containing a GFP-positive selection marker was used to infect immortalised porcine epithelial cells (EFN-R; CCLV-RIE 0089) grown on gold, holey-carbon coated electron microscopy grids and vitrified using a manual plunger with liquid ethane propane mixture (37%/63%).

#### WT PrV and HSV-1

EFN-R cells (CCLV-RIE 86), Vero cells (ATCC CCL-81) and immortalised human foreskin fibroblast cells (ATCC CRL-4001) were grown at 37°C with 5% CO_2_ in DMEM medium supplemented with 10% FBS and 1% Glutamax (Gibco). Gold grids with SiO_2_ film (Quantifoil, Au 200 mesh, R 1/4) were glow-discharged and coated with 20 μg ml^-1^ fibronectin solution for 30 min. Trypsinized cells were applied to grids and incubated for a minimum of 4 h and up to 12h. WT PrV (Kaplan) was added to the cells at MOI = 30 and incubated for 12 h. Separately, WT HSV-1 (strain 17) was added to the cells at MOI = 30 and incubated for 12-18 h. All samples were plunge-frozen either on Leica GP2 plunger or manual plunger in liquid ethane propane mixture (37%/63%).

### Lamellae Production using Dual-Beam cryoFIB-SEM

#### PrV-ΔUS3

Lamellae were milled using a dual-beam focused ion beam and scanning electron “Scios” microscope (Field Emission Industries, now Thermo Fisher Scientific, Oregon, USA) equipped with a Quorum PP3010 cryo transfer system. Milling was performed as outlined before^44^.

#### WT PrV and HSV

Vitrified HSV-1 and WT PrV infected samples were clipped in autogrids modified for FIB-milling and transferred to an Aquilos focused-ion beam scanning microscope (Thermo Fisher Scientific). Lamellae of approximate thickness between 100 nm and 250 nm were prepared using automated procedure with AutoTEM software (Thermo Fisher Scientific).

### Electron Cryo-Tomography Data Acquisition

#### PrV-ΔUS3

To probe the NEC-capsid interactions during herpesvirus nuclear egress, we targeted regions on the lamellae that contained enveloped nucleocapsids located within the lumen between the INM and ONM. At intermediate magnification (5,600x, nominal) it was possible to identify individual perinuclear vesicles and discern the characteristic electron-dense coat in between the nucleocapsid and membrane; these features were used to target tilt series collection at 61,000 x nominal magnification (3.55 Å/px).

CryoET data were acquired on lamella using a Tecnai G2 Polara transmission electron microscope (FEI) equipped with a field emission gun operated at 300kV, a GIF 2002 post-column energy filter (Gatan), and a Gatan K2 Summit Direct Electron Detector (Gatan, Pleasanton, CA) operating in counted mode. Tomographic tilt-series acquisition was performed under low-dose condition with a total of 30 tilt series from 27 lamellae. The tilt range used was from -60 to +60, with cumulative doses varying between ~ 80 - 200 e^-^Å^-2^. Data acquisition was performed using SerialEM^45^, with tilt-series acquired in a bidirectional manner using 3° tilt increments and defocus range of 3-6 μm.

#### WT PrV and HSV

Vitrified lamellae of WT HSV-1 or WT PrV infected cells were imaged on a Thermo Fisher Scientific Titan Krios TEM operating at 300 kV, equipped with a field emission gun (XFEG) and a Gatan Bioquantum energy filter with a slit of 20 eV and a Gatan K3 electron detector.

To screen lamellae for nuclear egress events, an intermediate magnification of 3600x was used. Tilt series were acquired using SerialEM software with cumulative dose of 100-120 e^-^Å^-2^, tilt range of -52 to +67 with the starting angle of 8° in dose symmetric manner^46^ and tilt increment of 3°. Defocus used ranged between 4-6 μm. Data was acquired at nominal magnifications of 42000x and 26000x and pixel sizes of either 2.15 Å px^-1^ for WT HSV^-1^ or 3.336 Å px^-1^ for WT PrV.

### Measurement of vesicle diameters

Measurements were performed if at least half the vesicle was contained within the tomogram (typically judged by at least half of the contained capsid being visible) and if primary envelopment was near or past completion. IMOD model points were manually placed on the vesicle membrane on the tomogram XY plane so that the cord between them intersected the rough centroid both in XY and XZ planes. In vesicles with elliptical cross sections, the smallest diameter was measured. The distance between the model points was then adjusted for twice the thickness of the membrane and the NEC lattice. Only vesicles containing at most one capsid were measured. Vesicles containing procapsid assemblies were classified as non-capsid vesicles as there were procapsids at various stages of assembly.

### Sub-Volume Averaging

Tomograms were reconstructed using IMOD Etomo or Aretomo^47^. WT PrV tilt series were scaled to pixel size 3.55 Å px^-1^, CTF corrected using IMOD ctfplotter and then reconstructed in Aretomo.

#### Spherical NEC

Sub-volume averaging was performed largely similar to that previously described for NEC vesicles in the absence of viral capsid^10^. Briefly, an initial reference was extracted from the tomogram which was centred on a single hexamer position within a perinuclear vesicle. This raw sub-volume was then used as the initial reference. 20 initial vesicles were modelled as a perfect sphere with a radius which was manually determined to be the rough diameter of a given vesicle in IMOD^48^. The model positions were sampled so that one point was roughly the space of an inter-heterodimer distance (10 nm). Subsequently, the model points were allowed to move in an iterative fashion using the sub-volume averaging software PEET^49^ with the sampling becoming progressively finer. Initial sub-volume averaging was performed on rescaled data until no improvement in resolution was observed and then the determined results applied to the original sampling, with sixfold symmetry applied, until the structure converged. This average was then used as a reference for the alignment of a further 37 vesicles modelled similarly as spheres, and 68 irregularly shaped vesicles and membrane patches containing NEC, which were picked using IMOD segmentation tools from 15 tomograms. These particles were aligned in a similar fashion to the original 20 vesicles, starting with data binned four times and gradually binning less and removing badly positioned, overlapping and poor scoring particles across five PEET runs. Sixfold symmetry was applied by creating duplicate particles with orientations rotated around each particle’s symmetry axis. A total of 22,596 particles were symmetrised, so that 135,576 particles were used in the final average. The Fourier shell correlation (FSC) for the NEC averages were calculated by splitting the final dataset in half, randomising the orientations of all particles and realigning separately. FSC plots were generated using Bsoft^50^ and the resolution taken at the 0.143 criterion. A soft-edge spherical mask (85 nm diameter) was applied to both half-maps.

For visualisation of ordered and disordered NEC regions, template matching was repeated on four vesicles, one of which was fully contained within the tomogram. Aligned particles were first cleaned by cross-correlation coefficient, and then manually cleaned to remove those that were outside hexagonally arrayed regions or those that were clearly misaligned (for example, centred on the membrane rather than the NEC layer). To determine the positions of particles missed by manual picking, the lattice formed by the particles was expanded to create new model points, by assuming C6 symmetry and an inter-hexamer distance of 110 nm, then aligned using a cylindrical mask with 22 nm diameter (roughly a single hexamer), and again cleaned manually and by cross-correlation coefficient. This process was repeated until the number of points remained roughly constant (Supplementary Fig. 8).

For HSV-1 NEC vesicles the same procedure was followed as described above using a spherical PrV NEC map as an initial reference binned four times. Four spherically shaped vesicles from four different tomograms were available for particle picking. A total of 1,421 particles were used, resulting in 8,526 particles in the final average after symmetrisation.

#### Nascent NEC

First, we established that the nascent NEC lattice resembles the spherical NEC lattice in perinuclear vesicles. This way, the spherical lattice map (with a high signal-noise ratio) could be used for template matching of the NEC in nascent budding sites. Model points were picked manually at three sites containing capsids in proximity of NEC patches, and their Y axes (normal to the membrane) were oriented to the nearest capsid centre. An initial reference was generated by averaging manually picked positions with random rotations around the Y axis. The average volume converged into a hexagonal lattice resembling the spherical NEC map. Subsequently, particles (generated using IMOD drawing tools) from 28 budding events in eight tomograms were aligned to an average volume of spherical NEC from perinuclear vesicles with volumes binned four times. Symmetry expansion was used to locate all hexagonal regions as described above (Supplementary Fig. 8). The resolution of the final map was not determined.

#### Tubular NEC

Four tubular NEC structures were first averaged independently. Initial references were obtained by averaging a small number of particles aligned to the cylinder symmetry axis and random rotations. These independently converged to a recognisable NEC lattice structure. Particle coverage was expanded from this initial particle seed using manually determined helical symmetry (-18.5°, -17°, and -12.16° twist and 22.7 Å, 22.7Å, and 71 Å rise, respectively). The four independent particle sets were then combined and averaged with progressively less binning. C2 symmetry was applied by adding particles rotated by 180° perpendicular to the helical symmetry axis. For FSC determination, particles were split after alignment with two times binned volumes and their rotations randomised by up to 5° along a randomly selected axis. The two halves were then aligned to the respective references obtained by particles with the randomised orientations.

#### Capsids

EMD-6907 was used as an initial reference with volumes binned eight times (57 Å Nyquist), followed by alignment of particles extracted at penton positions. These particles were split into two halves. Symmetry was applied by adding particles rotated by multiples of 72°. Alignment and averaging were performed with volumes binned four and two times and finally with unbinned volumes (3.55 Å Nyquist). The two half datasets were then combined, aligned, and filtered to their respective resolution at 0.143 FSC cutoff using Bsoft and an arbitrarily chosen B-factor.

### Data visualisation

Volumes were rendered using IMOD, Chimera, ChimeraX, Open3d or Matplotlib^51–54^. Manual segmentation of membranes was done using IMOD tools. Backplotting, the placement of average volumes into the original tomogram coordinate system using positions and orientations determined by subvolume averaging was performed using Python scripts based on TEMPy^55^.

### Rigid body fitting

To create pseudo-atomic models of the spherical and tubular NEC lattices, the crystal structure of PrV pUL31/34 (PDB 4Z3U) was fitted as rigid bodies into the relevant maps. An initial hexamer, together with six interacting heterodimers from neighbouring hexamers, were built by superposing the PrV structure atop the positions found in the symmetry expanded HSV-1 pUL31/34 crystal structure (PDB 4ZXS). The ChimeraX *Matchmaker* program was used for this superposition.

These twelve heterodimers were then fitted together as a single rigid body into the two NEC density maps, using ChimeraX ‘*Fit in Map*’. Each individual heterodimer was then fitted individually with the same program, using the previous fit as an initial position. Marker positions for the centre of mass of pUL31 residues 56-271 and pUL34 + pUL31 residues 18 - 55 were calculated using the ChimeraX ‘*measure*’ command, and the inter-marker distances using the distance dialogue box.

### Curvature analysis

Curvature analysis was performed by fitting a central surface normal and 2 principal curvatures to each central hexon and its neighbours within 426 Å (60 voxels x 7.1 Å voxel size) using scipy.optimize^56^ and NumPy^57^. The minimize function in scipy.optimize was used to minimize the root mean square error between a Gaussian surface (as defined by the values and directions of its two principal curvatures) to a set of localised points. The initial estimates for the directions were calculated using vectors orthogonal to the symmetry axis of the central particle. minimize was implemented using the SLSQP method, with constraints used to maintain normalised vectors and reasonable curvature values. Random perturbation of initial estimates and leave-one-out cross-validation were used to avoid overfitting and issues with local optima. This process was run for all subvolume particles from the spherical and tubular NEC lattices.

### Fluorescence microscopy

#### Cell culture

PK15 cells (DSMZ ACC 640) were cultivated in Dulbecco’s modified Eagle’s medium (Gibco) containing 10 % (v/v) foetal calf serum (Gibco) and kept at standard cell culture conditions (37° C, 95 % relative humidity, and 5 % CO2).

#### Viruses

PrV-mScarlet-UL25 was made by homologous recombination by transfecting PK15 cells with phenol-chloroform extracted WT PrV DNA (strain Kaplan^58^) and an 893 bp PCR-fragment coding for an insertion of mScarlet-I^59^ 5’ between amino acids 42 and 43 of pUL25 as previously reported^60^. PrV-mScarlet-UL25-ΔUS3 was generated similarly by homologous recombination through transfecting PK15 cells with phenol-chloroform extracted PrV DNA strain Kaplan ΔUS3^43^ but using a synthetic DNA construct (Biomatik) employing longer homologous sequences of 399 bp upstream and 573 bp downstream. After recombination, fluorescent viral clones were selected and underwent three rounds of plaque purification on PK15 cells.

#### Viral infections and sample preparation for microscopy

PK15 cells were seeded on 35 mm dishes (ibidi) and infected at a multiplicity of infection (MOI) of approximately 50, resulting in a mostly synchronised infection of all cells. After 1 h, the virus-containing media was removed, cells were washed with 1 ml of PBS, and new media was added. At indicated time points, cells were fixed for 15 min at room temperature with paraformaldehyde (4%) in PBS and washed three times with 1 ml of PBS. To counterstain nuclei, cells were incubated with Hoechst 33342 (Thermo Fisher) diluted to 16 μM in PBS and washed with PBS after 15 min.

#### Spinning disc fluorescence microscopy

Volumes of infected cells were acquired with a Nikon Eclipse Ti2 equipped with a Yokogawa CSU-W1, an Andor iXon Ultra DU-888U3 EMCCD camera, and an SR Apo TIRF AC 100xH objective (NA = 1.49) using 405 nm and 561 nm lasers with a quad filter (405/488/568/647) and 447/60 as well as 600/25 emission filters at a step size of 200 nm.

#### Single particle detection

Viral particle intensities were measured using Trackmate v6^61^ in FIJI^62^. Nuclear and cytosolic areas were marked as regions of interest (ROI), the expected blob diameter set to 0.4 μM, and the quality threshold set to 10.0. Total intensities were extracted from the resulting XML files and quantified with Origin.

#### Statistics and Reproducibility

All experiments were consistently reproducible across datasets and biological replicates to the extent that can be expected with observing individual events of biological processes. No statistical method was used to predetermine sample size and the investigators were not blinded to allocation during experiments and outcome assessment. Randomisation was used during subvolume averaging as indicated.

## Material availability

All cell lines (other than those available from public repositories) and all virus strains are available on request from corresponding author KG (expected response time 2 weeks).

## Extended Data

**Extended Data Table 1:** Comparison of key proteins, mentioned in this study, and their gene homologs from different species of alpha-, beta- and gamma-herpesviruses. Viruses from top to bottom: Pseudorabies Virus, Herpes Simplex Virus 1, Herpes Simplex Virus 2, Varicella Zoster Virus, Human Cytomegalovirus, Human Herpes Virus 6A, Kaposi’s Sarcoma-associated Herpes Virus, Epstein-Barr Virus.

VIRUS	US3	pUL31	pUL34	pUL25	pUL17	pUL36
**PrV**	**UL13**	**UL31**	**UL34**	**UL25**	**UL17**	**UL36**
**HSV1**	**UL13**	**UL31**	**UL34**	**UL25**	**UL17**	**UL36**
**HSV2**	**UL13**	**UL31**	**UL34**	**UL25**	**UL17**	**UL36**
**VZV**	**ORF47**	**ORF27**	**ORF24**	**ORF34**	**ORF42**	**ORF22**
**HCMV**	**UL97**	**UL53**	**UL50**	**UL77**	**UL93**	**UL48**
**HHV-6A**	**U69**	**U37**	**U34**	**U50**	**U64**	**U31**
**KHSV**	**ORF36**	**ORF69**	**ORF67**	**ORF19**	**ORF32**	**ORF64**
**EBV**	**BGLF4**	**BFLF2**	**BFRF1**	**BVRF1**	**BGLF1**	**BPLF1**

## Supplementary Material

Extended Data Figures

Source Data

Supplementary Information

Supplementary Video 1

Supplementary Video 2

Supplementary Video 3

Supplementary Video 4

## Data Availability

Maps for the following structures have been deposited in the Electron Microscopy Data Bank: PrV ΔUS3 capsids under accession codes EMD-17974 (cytosolic), EMD-17975 (perinuclear) and EMD-17976 (nuclear); PrV WT capsids under EMD-18479 (cytosolic) and EMD-18480 (nuclear); HSV-1 WT capsids under EMD-18481 (cytosolic), EMD-18482 (perinuclear) and EMD-18483 (nuclear); PrV ΔUS3 NEC under EMD-18474 (spherical form) and EMD-18473 (helical form); HSV-1 NEC under EMD-18484 (spherical form). All other data supporting the conclusions of this study can be found within the Article, extended data and Supplementary Information.

## Code availability

Custom scripts used for this study can be found at https://github.com/vojtaprazak/NEC
