# Supplementary material for: Molecular plasticity of herpesvirus nuclear egress analysed in situ
Source: Nat Microbiol. Author manuscript; Available in PMC 2024 Jul 9. (PMC7616147; doi:10.1038/s41564-024-01716-8)
Supplement: Extended Data Figures [file EMS197234-supplement-Extended_Data_Figures.docx]

**Extended Data Figures**

**
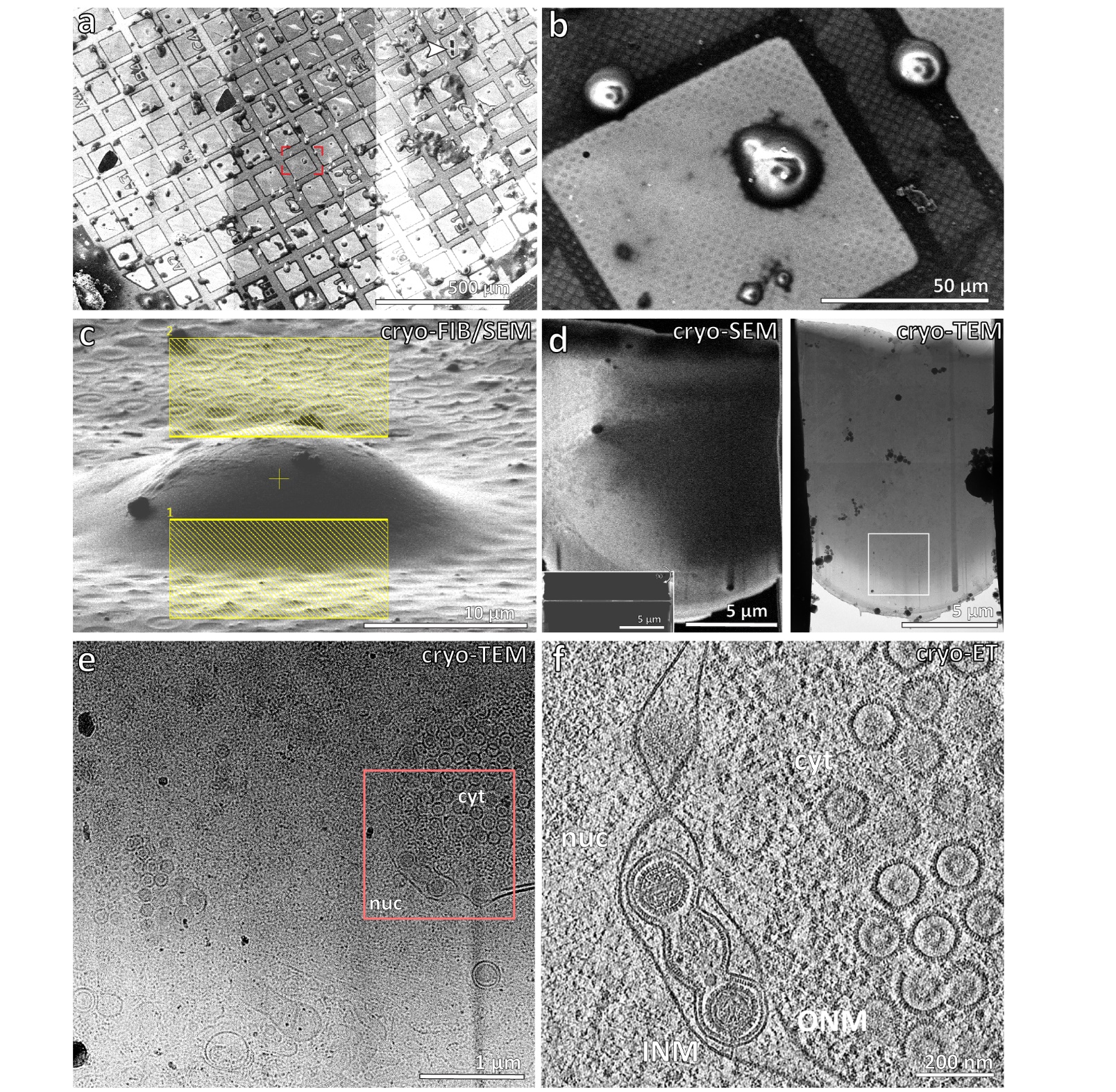
**

**Extended Data Fig. 1: Workflow for CryoFIB/SEM and CryoET.**  **a,** A low magnification SEM image of PrV-ΔUS3-infected porcine epithelial cells. **b,** A higher magnification image of the box shown in a. **c,** An oblique SEM view illuminated by the FIB with yellow boxes indicating the area above and below the cell for targeting with the focused ion beam. **d,** SEM image of thinned cellular section (lamella) from the top and side (inset). Transmission electron microscope (TEM) image is also shown (right), with target area shown in e highlighted by a white box. **e,** A TEM image shown at 9500x nominal magnification. Details of the cell are visible at this magnification, allowing targeting of regions of interest for higher magnification tomographic data collection (red box). Shown are representative samples of 113 lamella from 3 biological replicates. cyt = cytosol, nuc = nucleus. **f,** A tomographic slice of the region indicated in e) at 35000x magnification, nominal. ONM, outer nuclear membrane; INM, inner nuclear membrane.

**
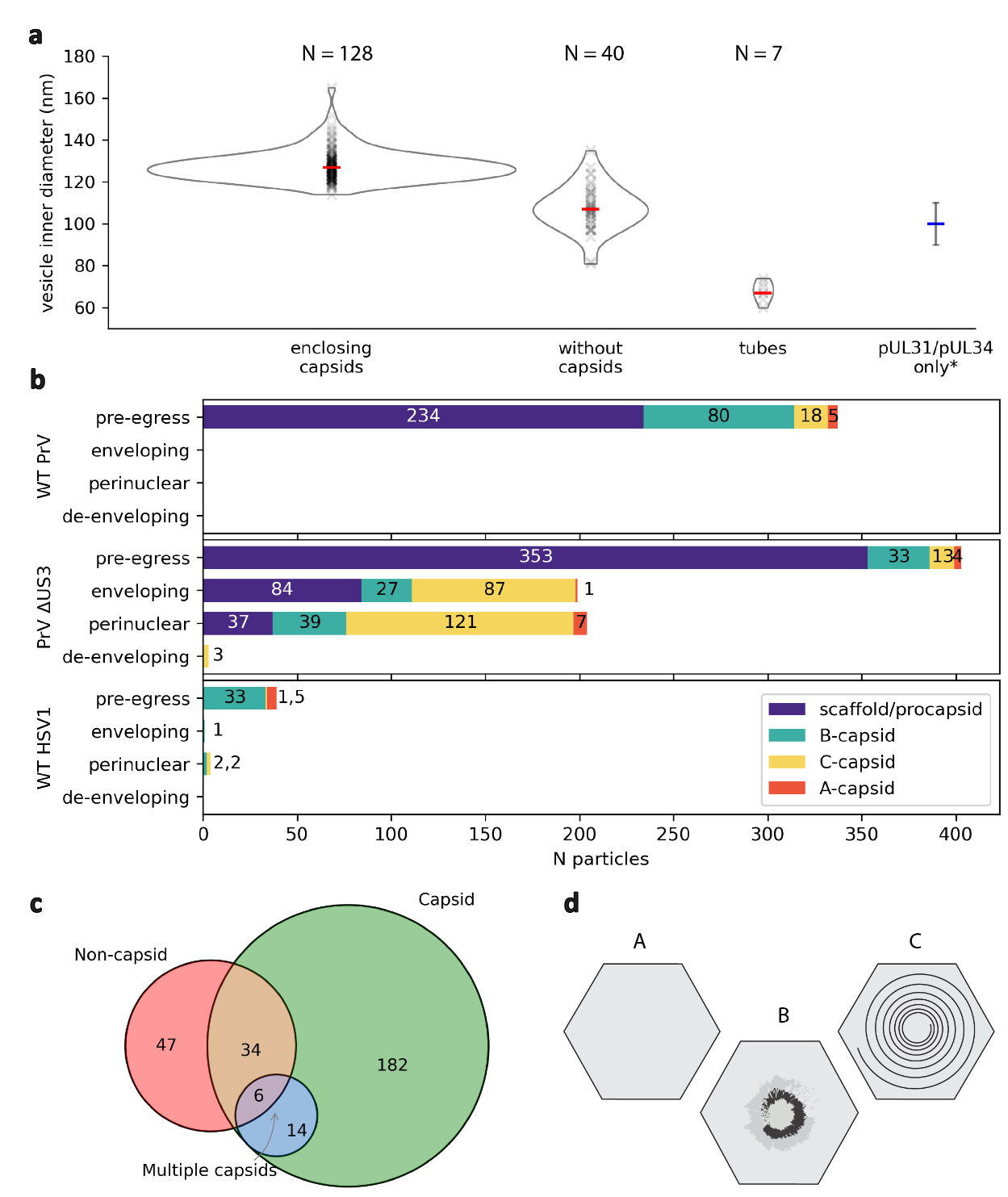
**

**Extended Data Fig 2: Quantification of nuclear egress events.** **a,** Size and curvature distribution of perinuclear vesicles**.** Shown are inner luminal diameters of individual vesicles (see Methods) with mean values represented by red bars. *The mean value and standard deviation of perinuclear vesicles obtained by expression pUL31/pUL34^9^ are shown for comparison. Vesicles (and the cargo within) were counted as perinuclear where envelopment was judged to be more than 50% completed (for example, Fig. 1c would be considered perinuclear). This is because a significant proportion of vesicles (~150 nm diameter) were only partially contained within the 150-250 nm thick tomograms (that is, cut off during FIB-milling). **b,** Quantification of capsid types observed during nuclear egress in porcine epithelial (PrV) and African Green monkey cells (HSV-1). **c,** Quantification of perinuclear vesicles. Classes were assigned to vesicles based on cargo type. In contrast to vesicle diameter measurements shown in **a**, vesicles containing procapsids were included in the capsid class, whereas scaffolds were counted as non-capsid cargo (note that there were 6 perinuclear procapsids). Procapsids contain capsid proteins some of which could be involved in the initiation of envelopment and therefore fit better in the “capsid” class. At the same time, all perinuclear procapsids were only partially assembled and consequently the perinuclear vesicles were smaller. **d,** Diagrams indicating capsid classification. A-capsids lack both nucleic acid and scaffold, B-capsids contain a scaffold, and C-capsids are capsids after completion of DNA packaging. Procapsids (not shown) are C-capsid precursors.

**
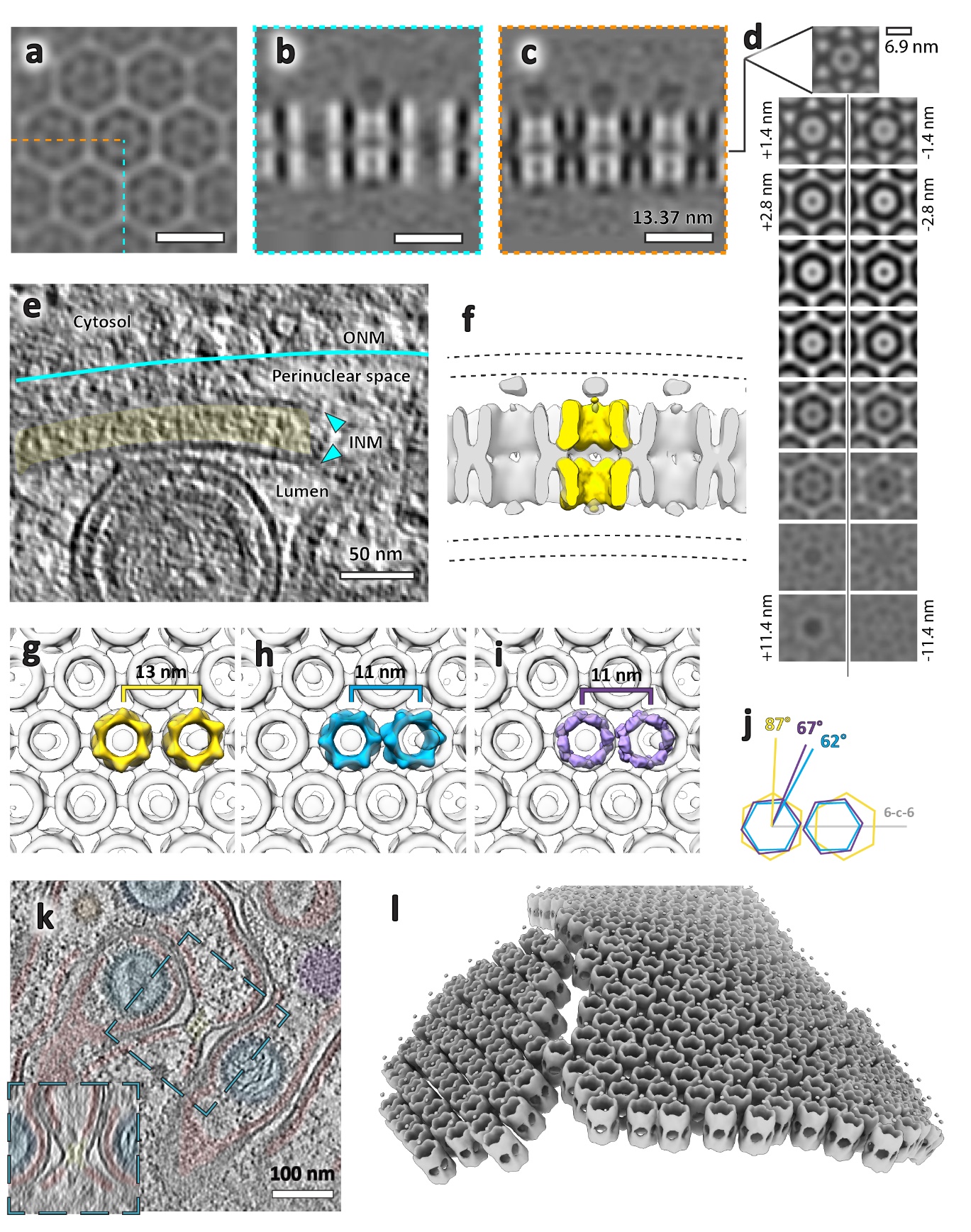
**

**Extended Data Fig. 3: Putative flat NEC double-layer lattice. a, b, c** Orthogonal section through average volume of lattice shown in Fig. 1f. There is no density for the membrane bilayers due to the majority of particles having their six-fold symmetry aligned with the tomogram Z axis (and the missing wedge). A total of 1944 C6 symmetrised particles were included in the average. **d,** Tangential sections through the volume at 1.4 nm intervals. **e,** A slice through the raw tomogram indicating the position of the inner nuclear membranes relative to the lattice. The membrane is not visible around the majority of the lattice layer due to its orientation to the tomogram missing wedge. The approximate position of the outer nuclear membrane was inferred from the exclusion zone of cytosolic components (ribosomes, intermediate filaments, microtubules). **f,** Two NEC hexamers segmented from the spherical lattice (Fig. 5, here shown in yellow) were fitted into one repeating unit of the lattice. In this orientation, pUL31 would form the interface between the two lattice layers. **g, h, i, j,** The hexamer centres in the double-layer lattice (yellow) are spaced 2 nm further apart and are rotated by approximately 20° to the 6-2-6 axis compared to spherical NEC (blue) and flat lattice derived from the HSV-1 crystal structure (purple). **k,** Slice through several adjacent type-1 NR, with the lumen of one of these zippered by putative head-head interacting NEC (yellow). Inset shows an orthogonal slice through the centre of the highlighted area. **i,** Plotback of individual symmetry units (dodecamers). There is a slight curvature to the lattice with a break in the middle, presumably to accommodate the tighter curvature of the underlying nucleoplasmic reticulum membrane.

**
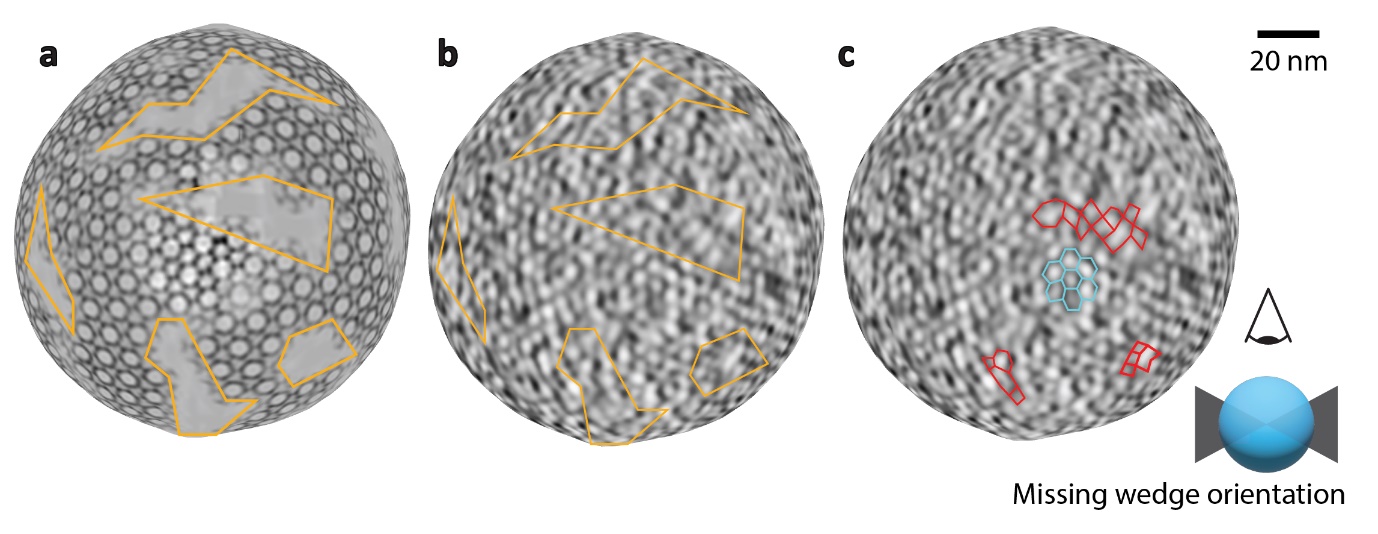
**

**Extended Data Fig. 4: Subvolume averaging generates a plausible model of NEC lattice order.** Shown is a top view of a single perinuclear vesicle (also in Figs. 2, 3, and Supplementary Fig. 8). The surface (generated and visualised using Open3D) was coloured with the intersecting voxel densities of either **a,** volume where an NEC average volume was backplotted using subvolume averaging particle positions or **b**, **c,** the original data. Orange lines highlight the same areas in a and b where particles were removed due to their relatively low cross correlation coefficient. A long-range hexagonal order is apparent outside these areas. Assessing the nature of disordered regions is more challenging. To guide the reader’s eye, some densities in putative disordered regions were highlighted with red lines. A single hexagonal region was highlighted in blue for comparison. Note: A direct interpretation of tomogram densities on this scale can be misleading and should be used with caution. This example is intended to highlight that there is likely NEC in the disordered regions. What the structure of this lattice may be is not clear.

**
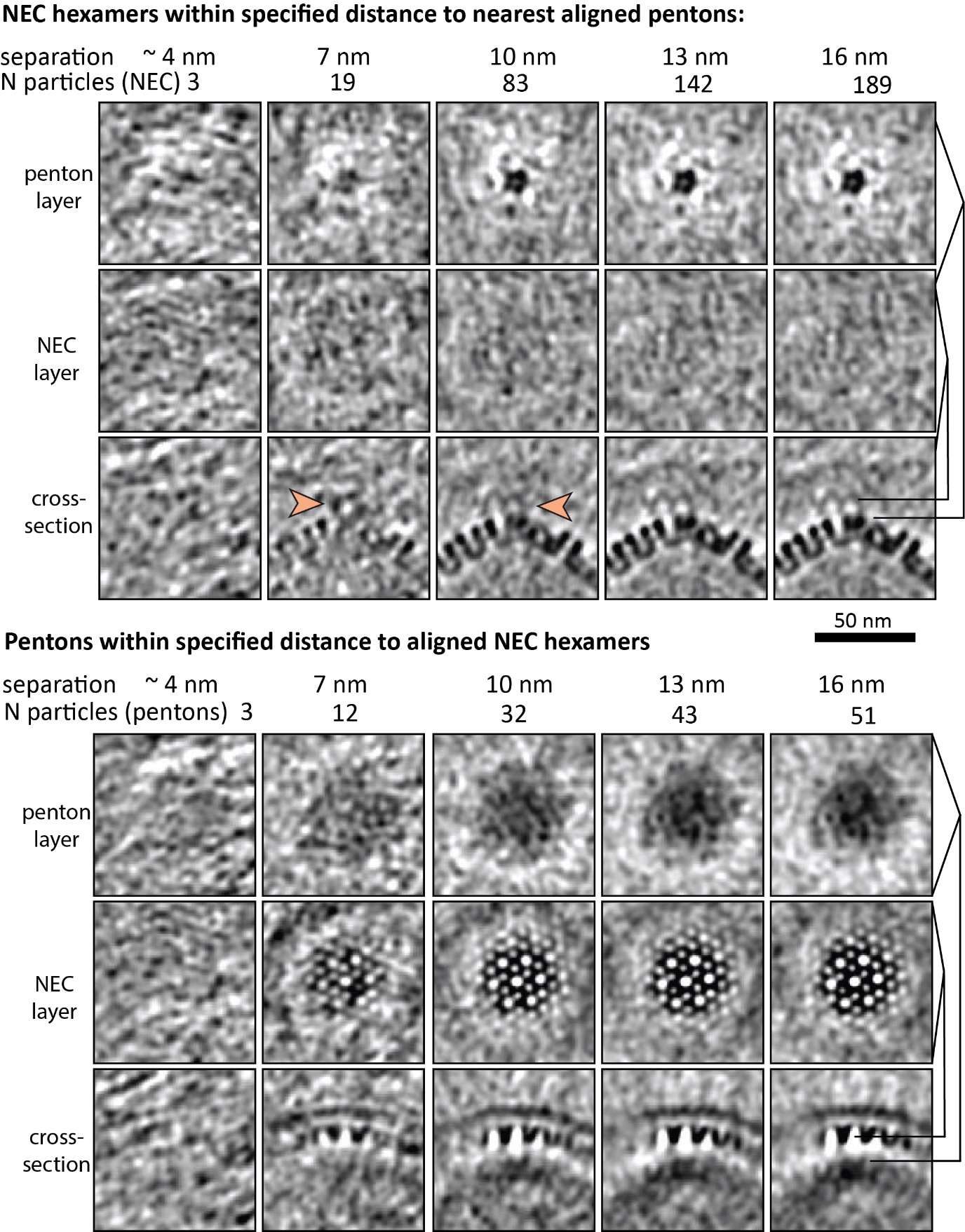
**

**Extended Data Fig. 5: Questionable connecting densities between nuclear capsids and budding NEC.** The NEC subvolumes were classified by the distance, to the nearest penton vertex (top panel) and vice versa (bottom panel). Each column shows sections through the resulting class average volume, with the maximum separation distance and the number of particles included in the average indicated above. All volumes were filtered using the same bandpass filter. The NEC layer is smeared in the penton-aligned averages and accordingly the capsids are smeared in the NEC-aligned averages, indicating that the two lattices are not aligned. There is a hint of a density originating from pentons closer than ~7 nm from the nearest NEC surface (orange arrows), but any interpretation of this would be highly questionable due to the small number of particles.

**
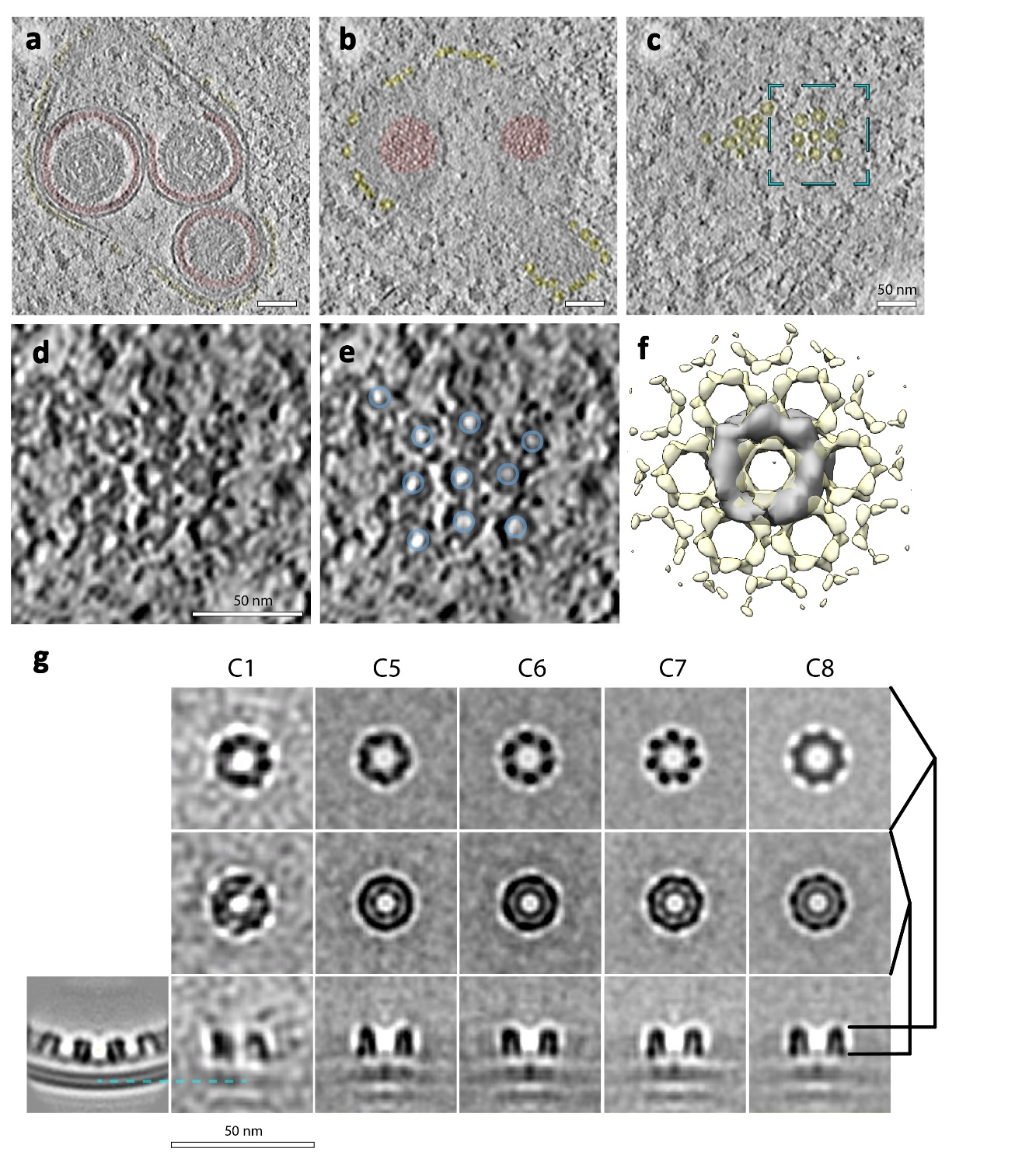
**

**Extended Data Fig. 6: Putative NEC coat on negatively curved surfaces. a, b, c,** Slices through the same nucleoplasmic reticulum at different depths showing the distribution of the putative NEC layer. **d, e,** An enlarged section of panel c, showing top views of ring-like structures (highlighted in blue in e). **f** Overlay of the surface representation of the average volume of 121 ring-like particles from two tomograms and the spherical NEC lattice. Each ring could plausibly accommodate two concentric layers of pUL31/34 dimers. Averaging a more exhaustive (but less stringently picked) set of negatively curved lattice particles did not converge (and is therefore not shown), suggesting a high degree of variability. Notably, the membrane was not included as an alignment feature. **g,** The thickness and distance to the membrane of this layer are consistent with the spherical NEC lattice. Sections through the ring average volume with different C symmetries applied. Visually, C7 is the best match to C1 but it is possible these structures have no strict symmetry, as suggested by d, e. Note that symmetrisation in this case means addition of subvolumes at defined rotations (for example 5 subvolumes with 60° degree increments for C6 symmetry). Alignment was performed after the addition of symmetry related particles. The bottom left-most panel is a section through the spherical NEC lattice.

**
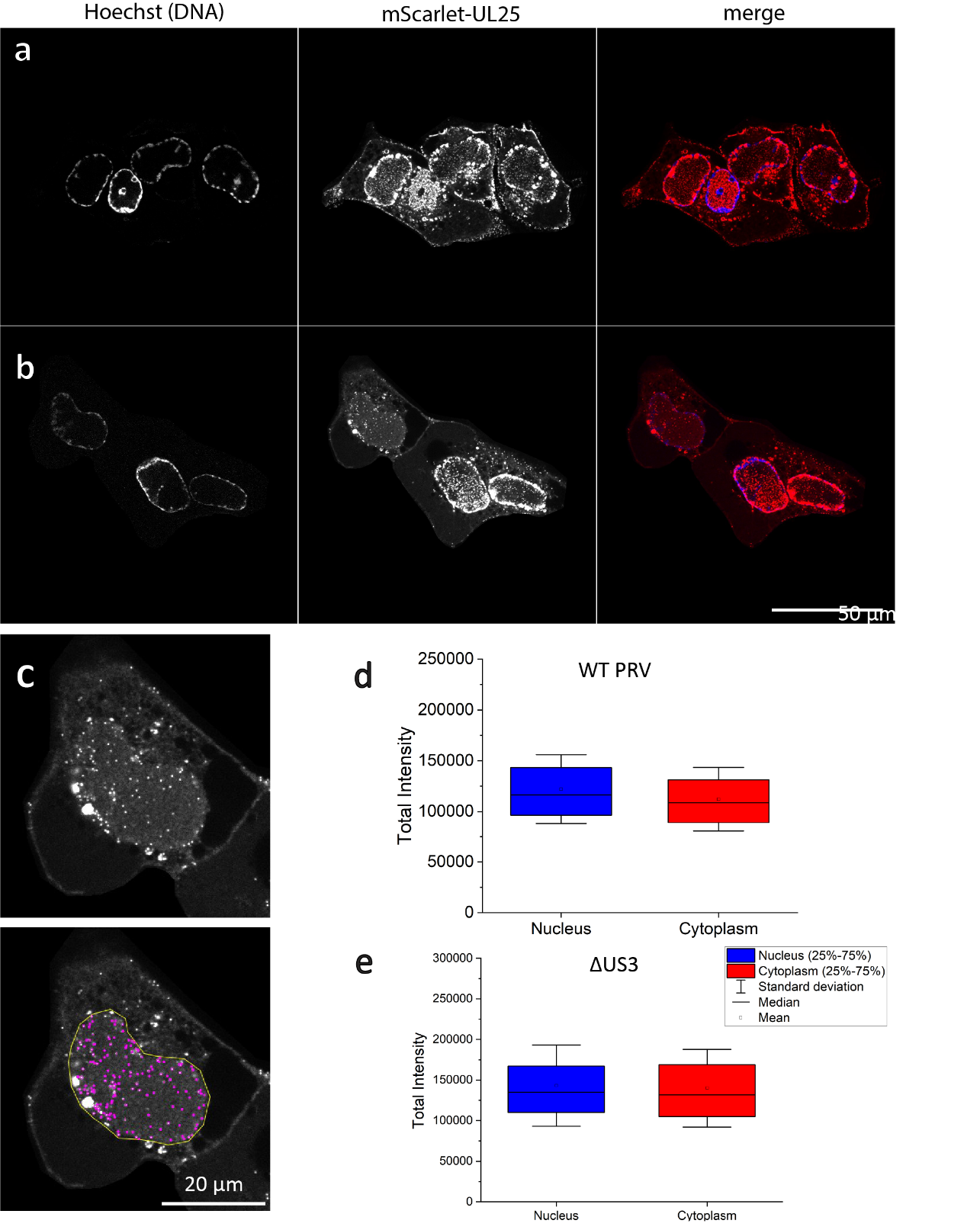
**

**Extended Data Fig. 7: Localization of individual nuclear and cytoplasmic mScarlet-UL25 labelled capsids. a,** PK15 cells were infected with PrV-mScarlet-UL25 or **b,** PrV-mScarlet-UL25-∆US3 fixed at 7 or 10 hpi, respectively, and imaged using spinning disc microscopy. UL25-mScarlet (red); DNA-Hoechst (blue). One plane of the acquired volume is shown. **c,** Viral particles were detected in the 3D volumes using Trackmate in FIJI with an expected blob diameter of 0.4 microns and the quality threshold set to 10.0. **c,** Fluorescent signal in a single plane of a PK15 cell infected with PrV-mScarlet-UL25-∆US3 and fixed 10 hpi (top) and projection of all detected single particles (purple) of the volume in a nuclear ROI (yellow) onto one plane (bottom). **d, e,** The FIJI plugin Trackmate was used to detect and measure individual virus particle fluorescent intensities. For each condition, the total intensity of more than 4,000 particles was quantified and detected in more than 30 different cells, all using a single biological replicate.

**
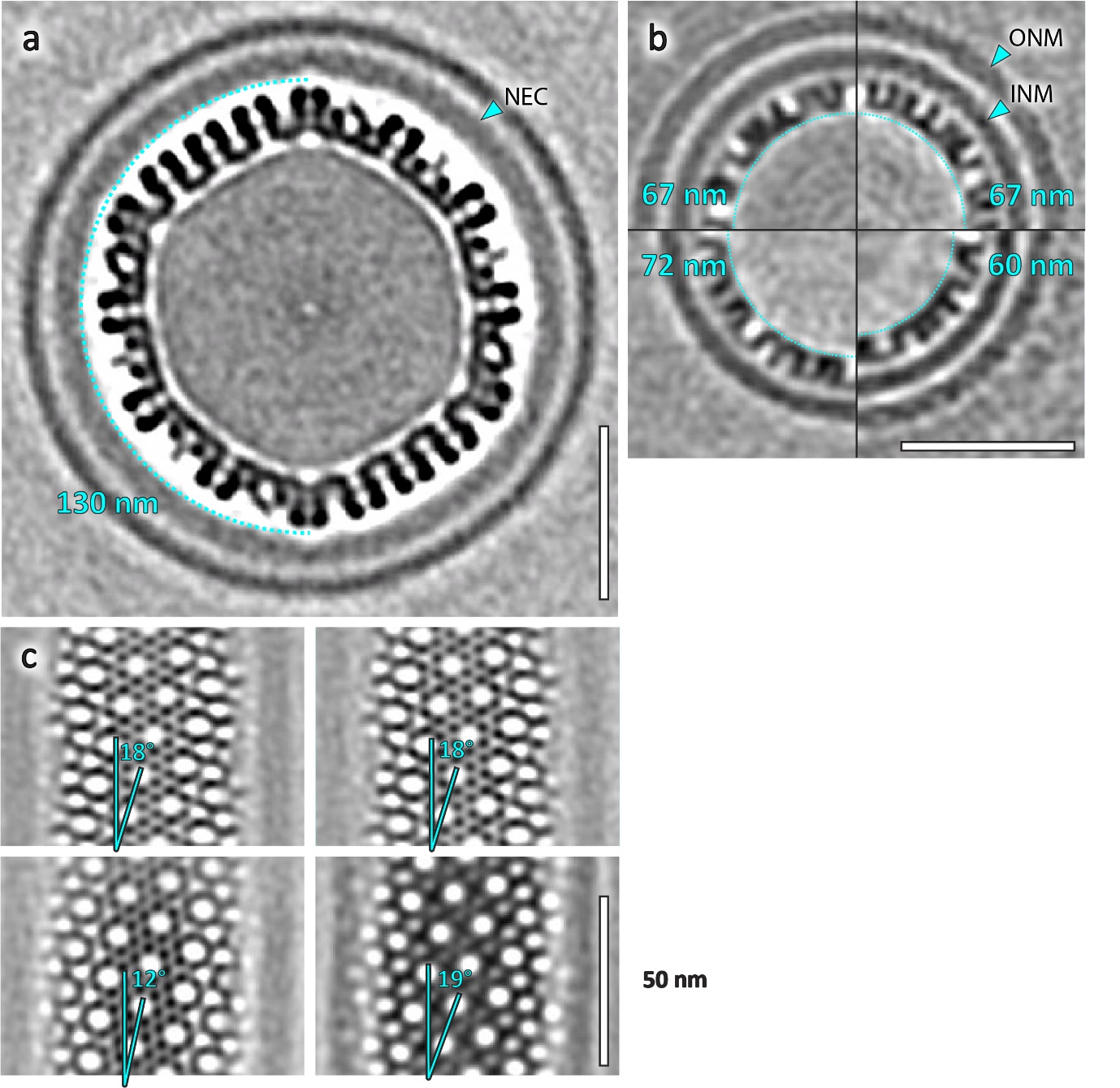
**

**Extended Data Fig. 8:** **PrV nuclear egress complex forms tubes with helical symmetry. a**, **b,** Comparison of the NEC curvature in perinuclear vesicles and tubes. **b,** Shown are sections through the average volumes of four tubes, with the respective diameters indicated in cyan. The NEC tubes have substantially smaller luminal cavities compared to spherical NEC vesicles. Notably, the two separate tubes with matching diameters and helical parameters were located within the same nucleoplasmic reticulum and may have originated from the same assembly. **c,** Tubular NEC forms tubes with different helical parameters, indicating a flexibility in the direction of largest curvature. Highlighted is the angle of the 6-2-6 axis to the helical symmetry axis. Scale bars indicate 50 nm.

**
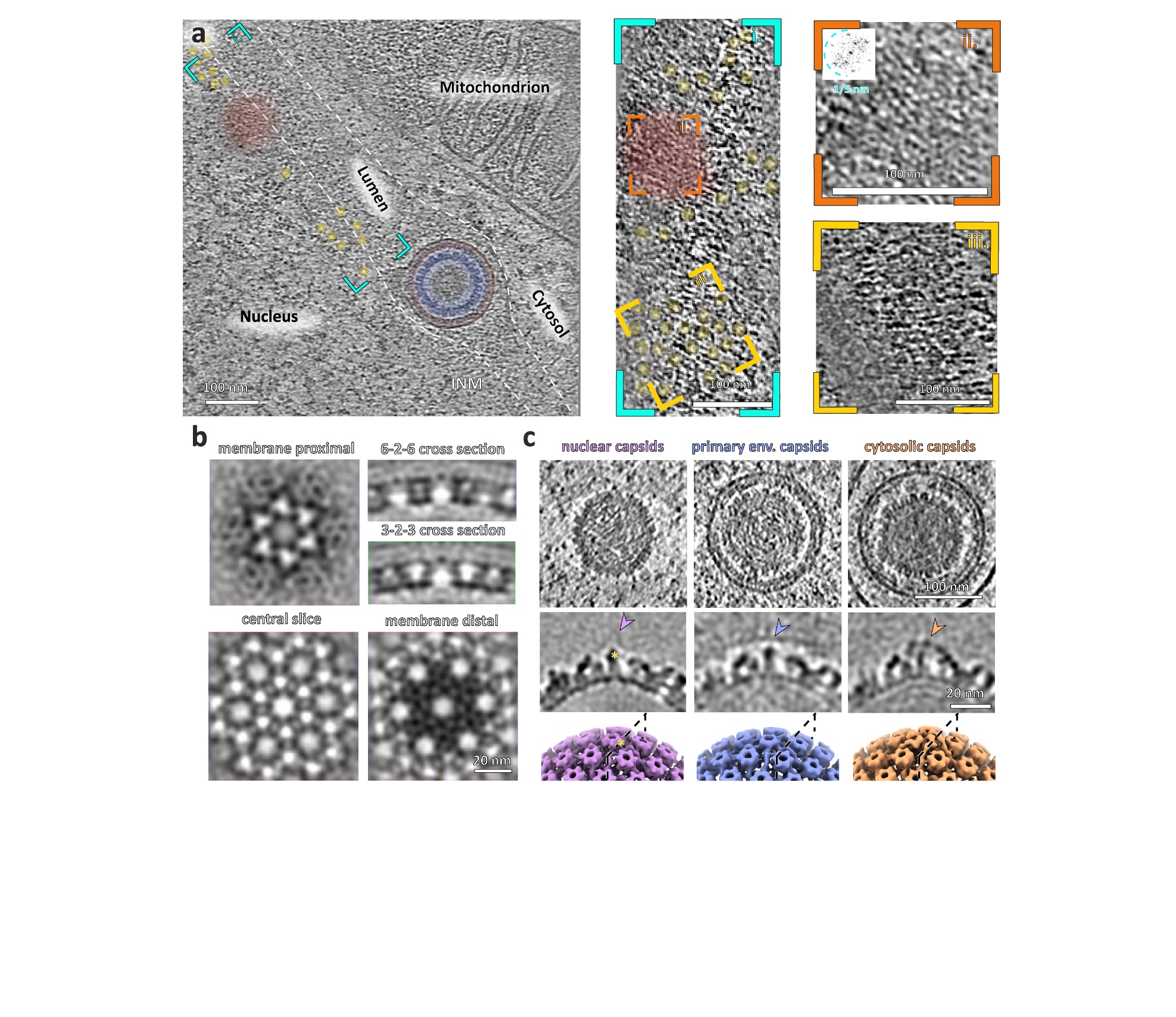
**

**Extended Data Fig. 9: NEC in HSV1**. **a,** Slice through a tomogram depicting primary enveloped nucleocapsid egressing to the cytosol. The NEC coat of WT HSV-1 (red) follows the same pattern as that of ΔUS3 PrV and could be identified on the INM and in perinuclear vesicles. Likewise ring-like structures similar to those in PrV (yellow in i. and iii., Fig. 3c and Extended Data Fig. 6) was identified near the hexagonal NEC lattice (red in i. and iii.). Red area in the inset ii. shows budding NEC. **b,** HSV1 NEC crystal structure (PDB 4ZXS) fitted into the subvolume average of HSV-1 NEC from perinuclear vesicles. ONM, outer nuclear membrane; INM, inner nuclear membrane. **c,** Raw tomographic slices through C-capsids of WT HSV-1 in indicated subcellular locations. **d,** Slices through the average volumes of C-capsids (middle) and their surface representations (bottom). Arrowheads indicate the position of an additional density present in the cytosolic capsids. 15 tomograms from 5 preparations were used.

**
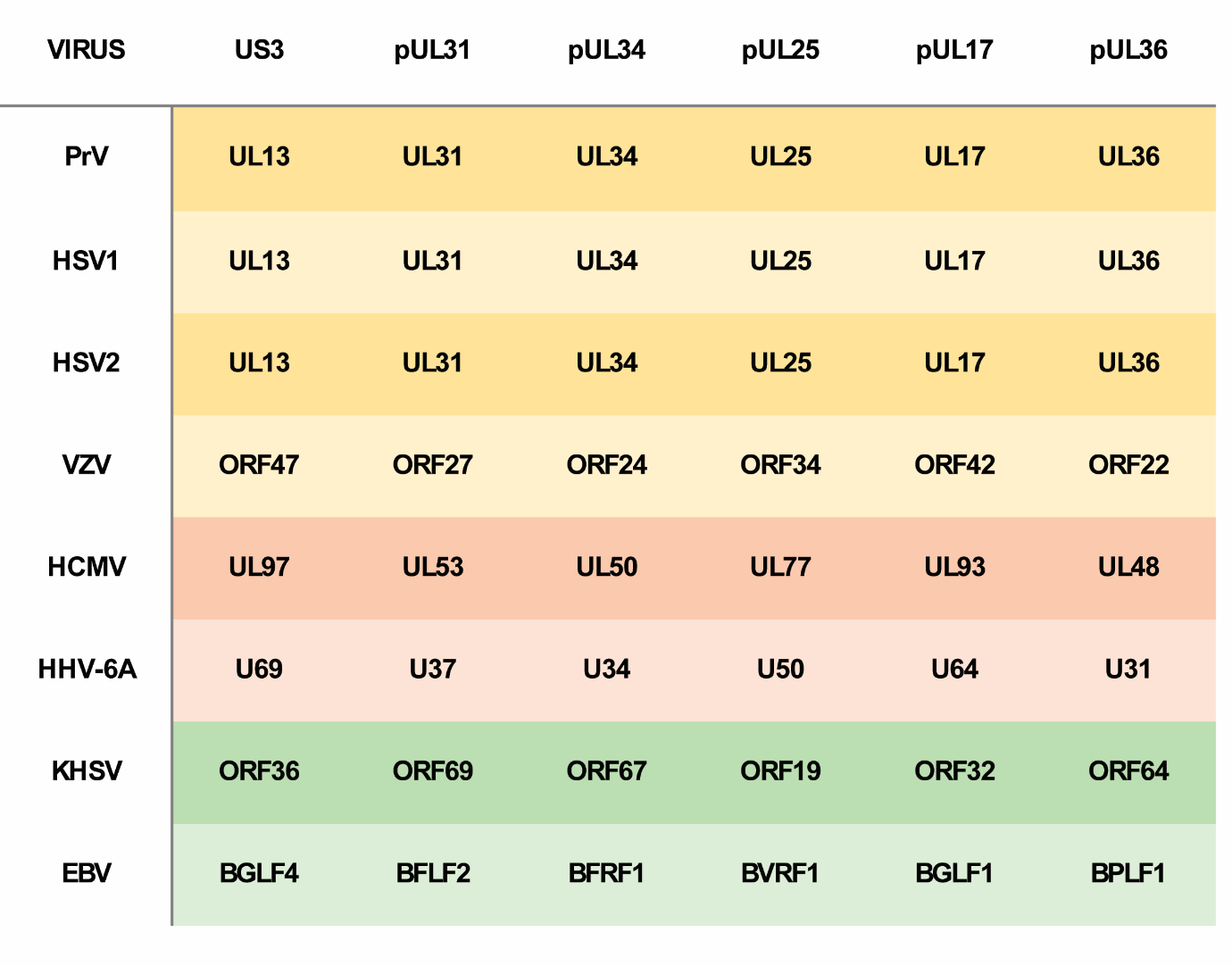
**

**Extended Data Table 1:** Comparison of key proteins, mentioned in this study, and their gene homologs from different species of alpha-, beta- and gamma- herpesviruses. Viruses from top to bottom: Pseudorabies Virus, Herpes Simplex Virus 1, Herpes Simplex Virus 2, Varicella Zoster Virus, Human Cytomegalovirus, Human Herpes Virus 6A, Kaposi’s Sarcoma-associated Herpes Virus, Epstein-Barr Virus.
