# Supplementary material for: Molecular plasticity of herpesvirus nuclear egress analysed in situ
Source: Nat Microbiol. Author manuscript; Available in PMC 2024 Jul 9. (PMC7616147; doi:10.1038/s41564-024-01716-8)
Supplement: Supplementary Information [file EMS197234-supplement-Supplementary_Information.docx]

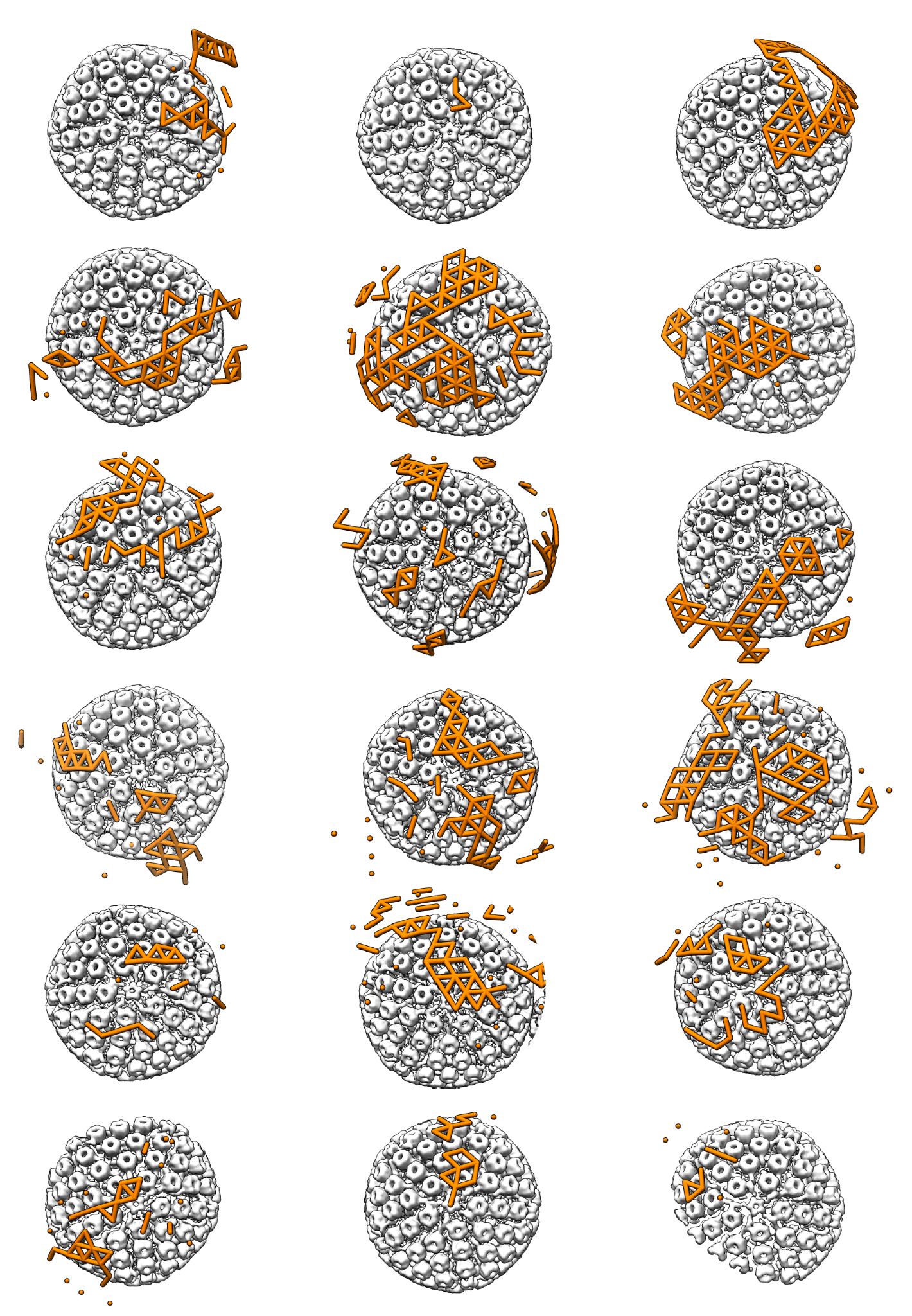


**Supplementary Fig. 1: 3D overview of putative budding events.** Capsid volumes are aligned with one penton vertex facing the viewer. Orange bars connect NEC hexamer centres separated by less than 12 nm (the spacing between hexamers in the spherical lattice).


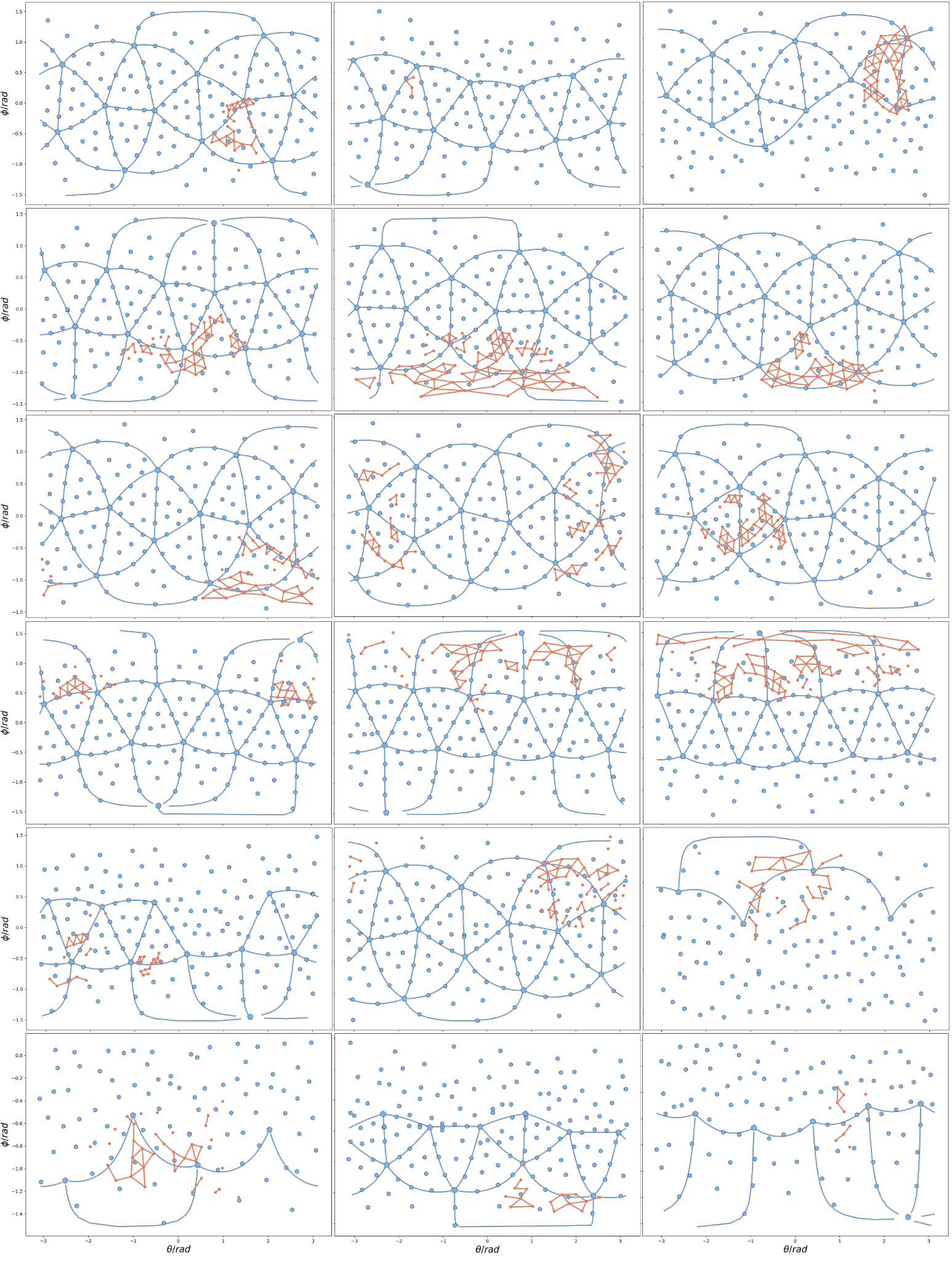


**Supplementary Fig. 2: Radial coordinate overview of putative budding events**. These events are the same as those shown in Fig. S5, but represented in radial coordinate space with the capsid centre as the origin. Blue lines represent icosahedral edges, pentagons represent penton vertices, and circles represent hexons. Some hexon and penton positions are obviously misaligned. This is due to some capsids being only partially contained within the lamella. Orange lines connect NEC hexamer centres.


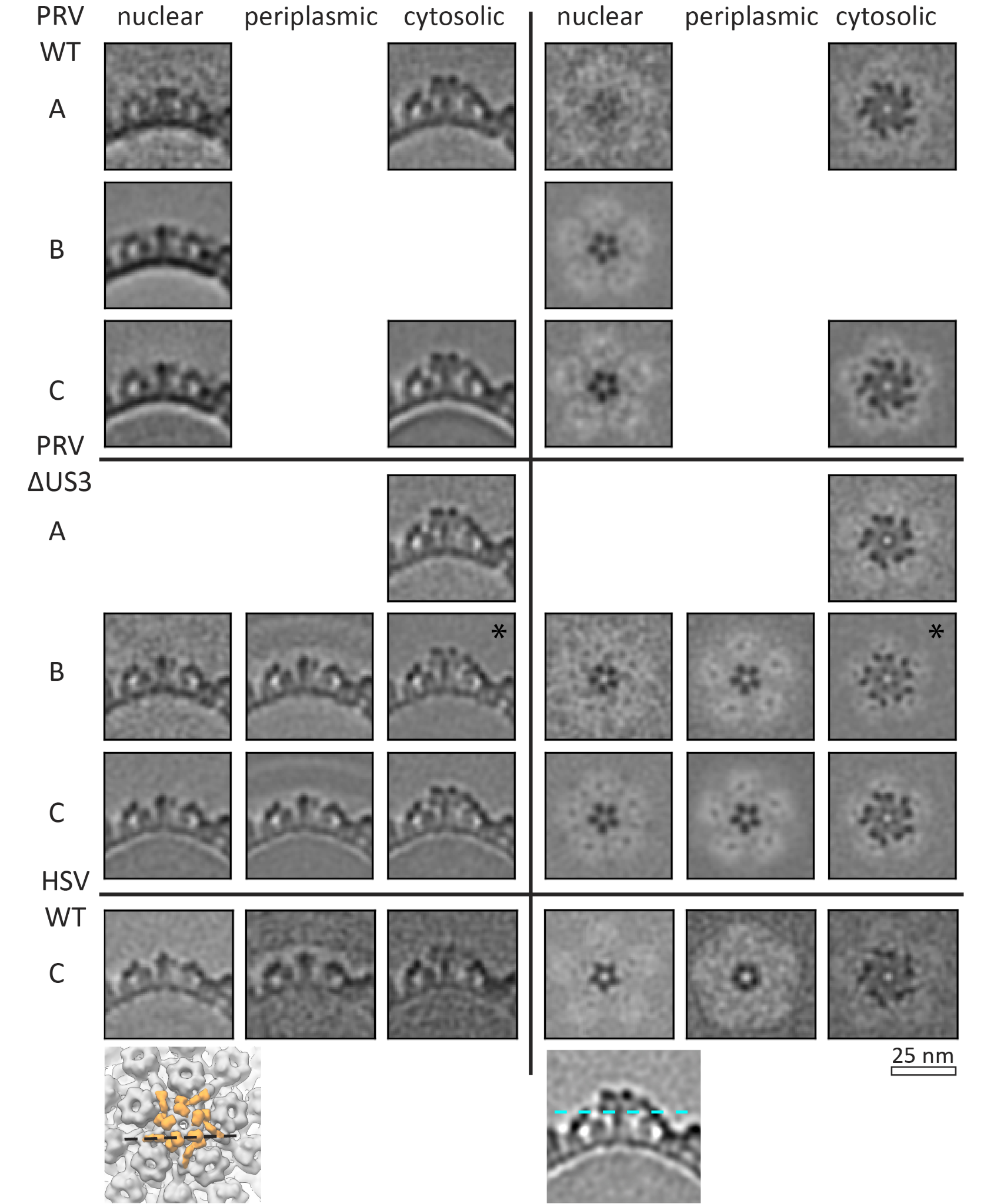


**Supplementary Fig. 3: Overview of capsid volumes.** Shown are normalised average volumes of A-, B-, and C- capsids located in the nucleoplasm, perinuclear vesicles, and cytosol, respectively (also in Fig. 4). *The perinuclear B-capsid class consist of particles with visible scaffolds or partially spooled genomes. The dashed lines in bottom row images represent sectioning planes of the respective columns.


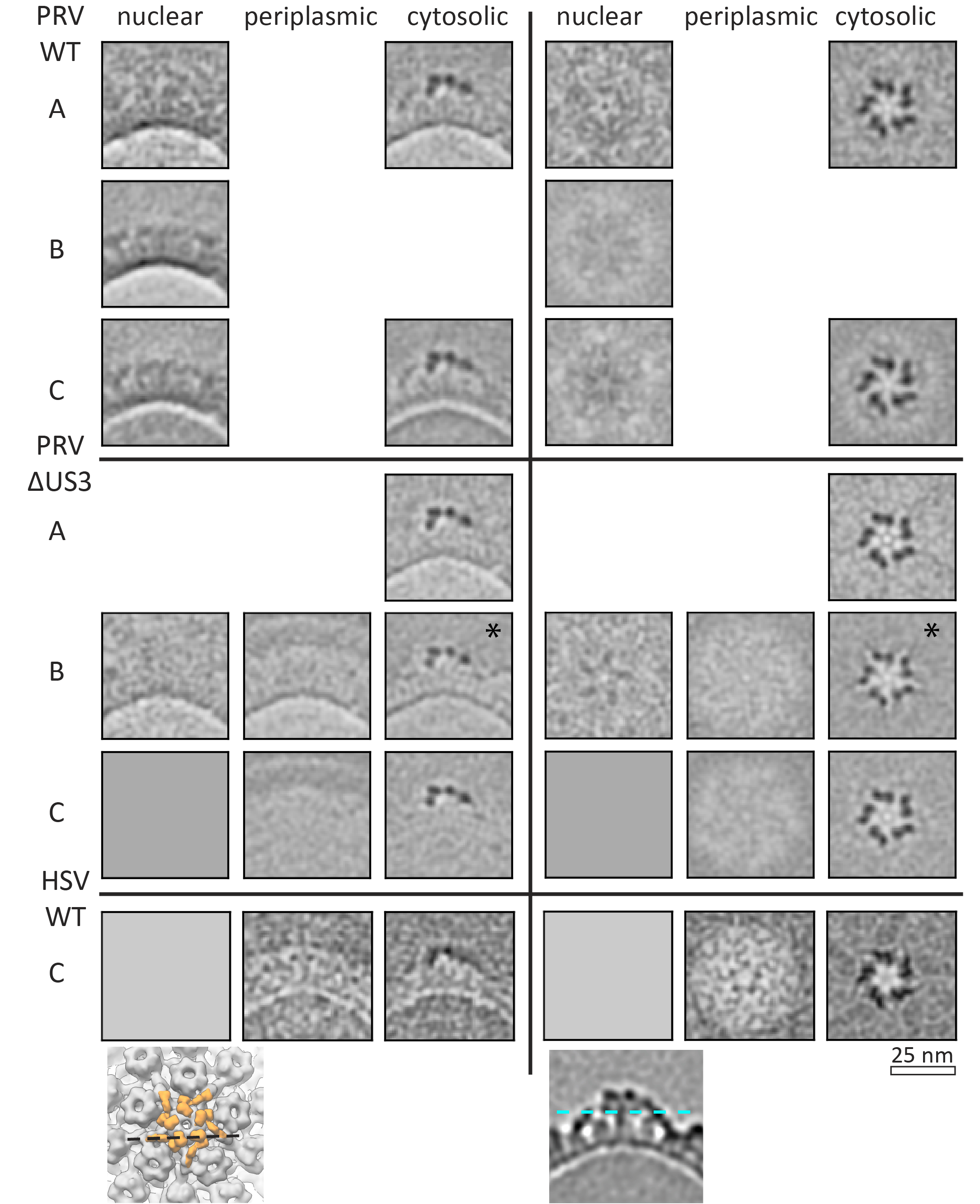


**Supplementary Fig. 4: Overview of normalised capsid volume differences.** PrV-ΔUS3 nuclear C-capsid volume was subtracted from WT PrV and PrV-ΔUS3 A-, B-, and C-capsid volumes derived from particles located in the nucleoplasm, perinuclear vesicles, and cytosol, respectively. HSV1 nuclear C-capsid volume was subtracted from HSV perinuclear and nuclear C-capsids. *The perinuclear B-capsid class consist of particles with visible scaffolds or partially spooled genomes. The dashed lines in bottom row images represent sectioning planes of the respective columns.


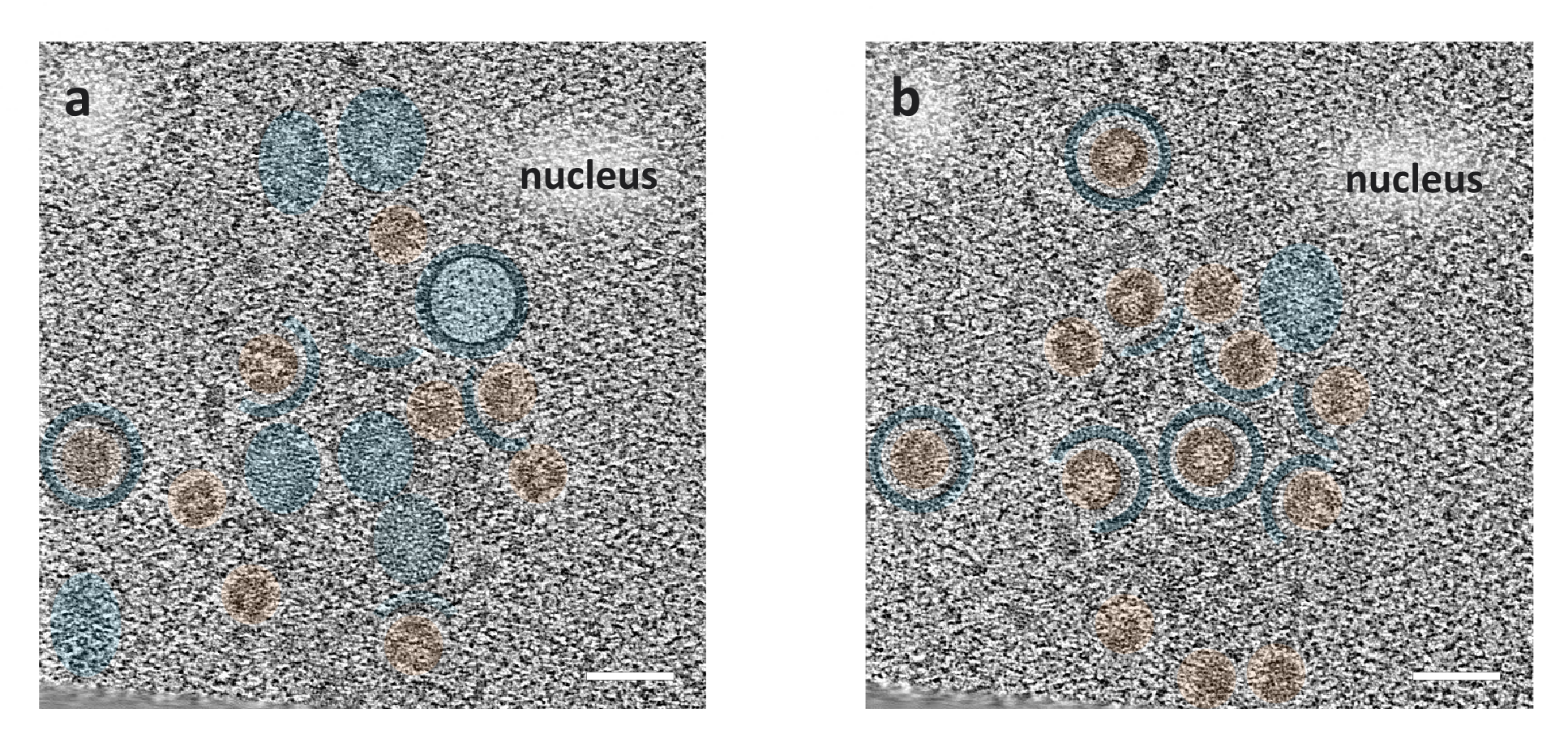


**Supplementary Fig. 5: Nucleoplasm of Vero cells infected with WT PrV at 12 h post infection.** Shown are tomogram slices separated by ~25 nm in Z. Depicted capsid assembly sites are similar to those in PrV-ΔUS3 (Fig. 1b). Capsid components are highlighted in blue, scaffolds in orange. Scale bars represent 100 nm. In total, 34 tomograms from 2 preparations were used for WT PrV analysis.


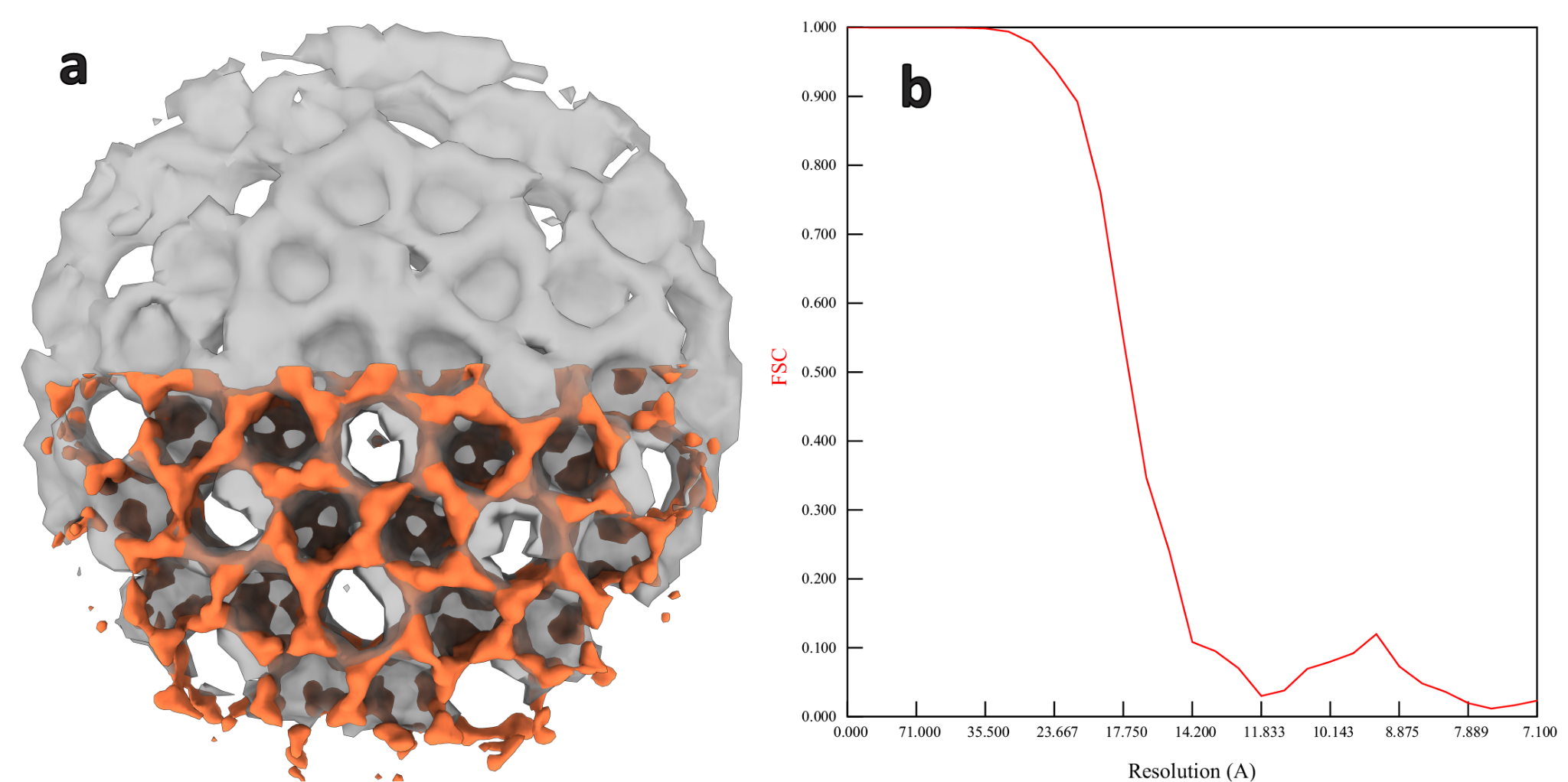


**Supplementary Fig. 6: PrV-ΔUS3 NEC matches the structure of pUL31/34-GFP. a,** Comparison of EMD-3215 (grey) and the map of spherical NEC determined in this study (orange). **b,** Gold-standard Fourier-shell correlation of the latter map.


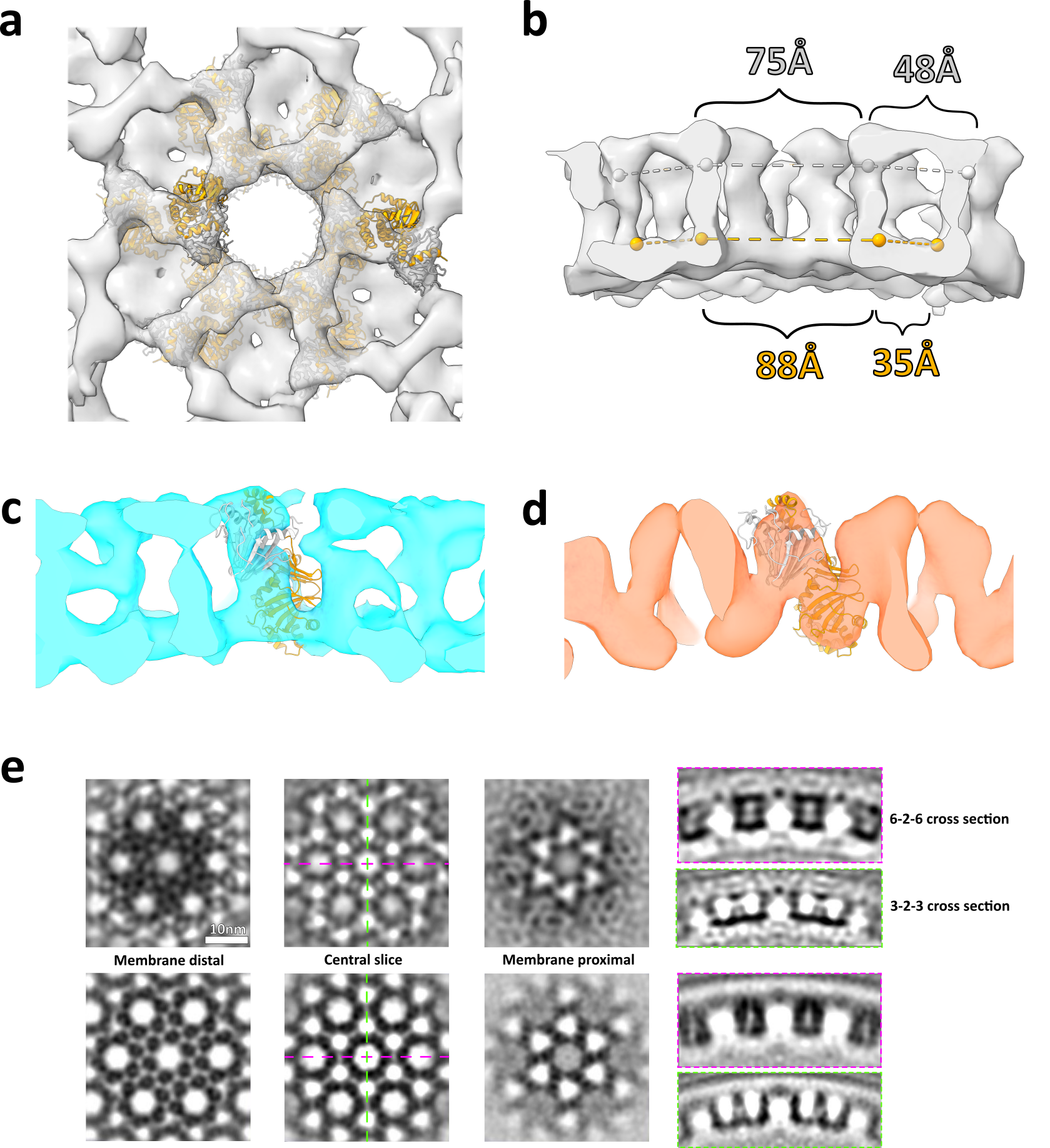


**Supplementary Fig. 7: Fitting of HSV1 pUL31/34 crystal structure into HSV1 NEC map.** The same fitting process was applied as seen in Figure 8 and the Materials and Methods - Rigid Body Fitting section. However, the fit led to significant clashes between inter-hexamer dimers, and is therefore even less reliable than the similar PrV analysis. **a,** View of the lattice with membrane proximal region facing the viewer. Subvolume average is shown as grey isosurface, pUL31 as orange ribbon and pUL34 as light grey ribbon. **b,** Distances between centre of mass markers on opposing sides of the HSV-1 NEC hexamers. Light grey markers represent centre of mass of pUL34 and arm of pUL31 (residues 56-84), orange markers represent centre of mass of the remainder of pUL31. **c** Fit of single HSV-1 pUL31/34 heterodimer into subvolume average of HSV-1 NEC. **d,** Fit of single HSV-1 pUL31/34 heterodimer into subvolume average of PrV NEC. **e,** Slices through the electron densities of the HSV-1 NEC (top) and the PrV NEC (bottom) subvolume averaging structures. From left to right: membrane distal slice, representing the pUL34 part of each heterodimer; central slice, with the green dotted line along the 6-2-6 direction and the magenta line through the 3-2-3 direction of the lattice; membrane proximal slice, representing pUL34 densities; and finally cross sections through the 6-2-6 and 3-2-3 directions of the NEC.

**
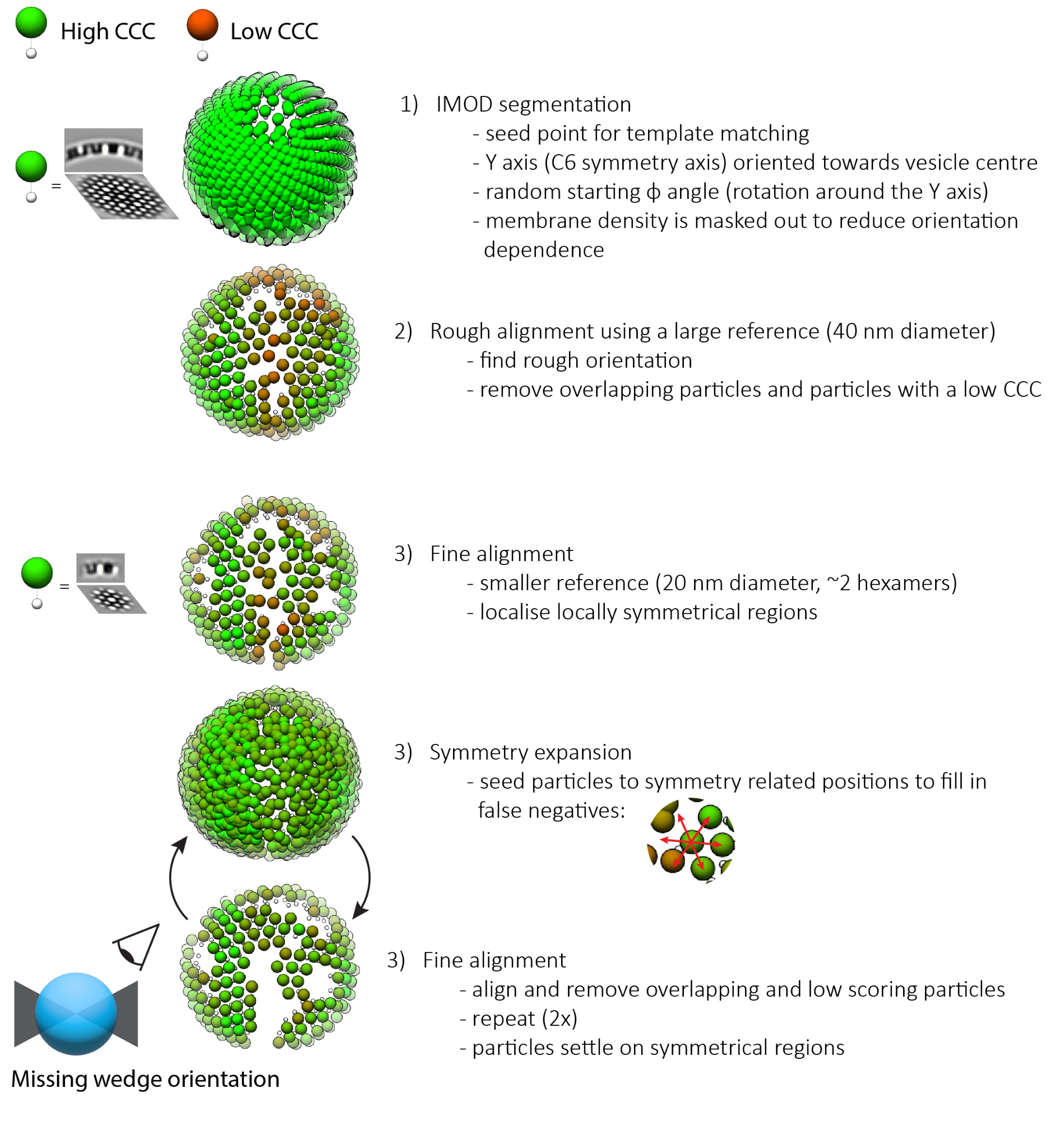
**

**Supplementary Fig. 8: Workflow of template matching and symmetry expansion.** Shown is a single perinuclear vesicle, with particle points represented as pins.

| **PrV capsid, WT** |  | **nuclear** | **perinuclear** | **cytosolic** |
| --- | --- | --- | --- | --- |
| **A** | N particles | 410 | N/A | 325 |
|  | FSC 0.143 | 40 Å | - | 27 Å |
|  | EMD | 18480 | - | 18479 |
| **B** | N particles | 5445 | N/A | N/A |
|  | FSC 0.143 | 22 Å | - | - |
|  | EMD | 18480 | - | - |
| **C** | N particles | 1920 | N/A | 3445 |
|  | FSC 0.143 | 29 Å | - | 26 Å |
|  | EMD | 18480 | - | 18479 |
| **PrV capsid, ΔUS3** |  | **nuclear** | **perinuclear** | **cytosolic** |
| **A** | N particles | N/A | N/A | 410 |
|  | FSC 0.143 | - | - | 34 Å |
|  | EMD | - | - | 17974 |
| **B** | N particles | 295 | 1030* | 735 |
|  | FSC 0.143 | 31 Å | 29 Å* | 22 Å |
|  | EMD | 17976 | 17975* | 17974 |
| **C** | N particles | 1665 | 3950 | 1065 |
|  | FSC 0.143 | 24 Å | 23 Å | 31 Å |
|  | EMD | 17976 | 17975 | 17974 |
| **HSV capsid, WT** |  | **nuclear** | **perinuclear** | **cytosolic** |
| **C** | N particles | 1170 | 205* | 450 |
|  | FSC 0.143 | 30 Å | 38 Å | 35 Å |
|  | EMD | 18483 | 18482 | 18481 |
|  |  |  |  |  |
|  |  | **Spherical PrV NEC, ΔUS3** | **Spherical HSV NEC, WT** | **Helical PrV NEC, ΔUS3** |
|  | N particles | 135,576 | 8526 | 4247 |
|  | FSC 0.143 | 14 Å | 27 Å | 21 Å |
|  | EMD | 18474 | 18484 | 18473 |

**Supplemental Table 1:** Maps presented in this study. *-mixed type of capsids.

**Sequence data**

PrV-mScarlet-UL25 was made by homologous recombination by transfecting PK15 cells with phenol-chloroform extracted wildtype PrV DNA (strain Kaplan (Kaplan and Vatter (1959) and an 893bp PCR-fragment coding for an insertion of mScarlet-I (Bindels et al., 2017) 5’ between amino acids 42 and 43 of UL25 as previously reported (Bohannon et al., 2013).

Used primer sequences are:

Forward Primer: 5’-GCC GCT GCG ATC CCG GGC AGC GCG CGC CAC TTC ATC GCG CCC CCG TTC CCC GTG GGC TTC TGG GCC CGC CCG GGC TTC AGC GAG GGC CTC GAC GCG CGC GTG AGC AAG GGC GAG GCA GTG-3’

Reverse Primer: 5’-TGC TCG TCC ACC TCG GCC TCG AGG CGC GCG CCC GCG GCC ATG GCG TTG TCC AGC GCC GCG GCG GCC GCG CGG CGG CGC GCG TTC GCG TGC GCC AGC GCG AGC TTG TAC AGC TCG TCC ATG-3’

PrV-mScarlet-UL25-∆US3 was generated similarly by homologous recombination through transfecting PK15 cells with phenol-chloroform extracted PrV DNA strain Kaplan ∆US3 (Klupp et al., 2001) but using a synthetic DNA construct (Biomatik, Canada) employing longer homologues sequences of 399 bp upstream and 573 bp downstream. After recombination, fluorescent viral clones were selected and underwent three rounds of plaque purification on PK15 cells.
